# Specific cannabinoids revive adaptive immunity by reversing immune evasion mechanisms in metastatic tumours

**DOI:** 10.3389/fimmu.2022.982082

**Published:** 2023-02-22

**Authors:** Sarah Dada, Samantha L. S. Ellis, Christi Wood, Lilian L. Nohara, Carola Dreier, Nicolas H. Garcia, Iryna Saranchova, Lonna Munro, Cheryl G. Pfeifer, Brett A. Eyford, Suresh Kari, Emmanuel Garrovillas, Giorgia Caspani, Eliana Al Haddad, Patrick W. Gray, Tunc Morova, Nathan A. Lack, Raymond J. Andersen, Larry Tjoelker, Wilfred A. Jefferies

**Affiliations:** ^1^ Michael Smith Laboratories, University of British Columbia, Vancouver, BC, Canada; ^2^ Vancouver Prostate Centre, Vancouver Coastal Health Research Institute, Vancouver, BC, Canada; ^3^ Centre for Blood Research, University of British Columbia, Vancouver, BC, Canada; ^4^ The Djavad Mowafaghian Centre for Brain Health, University of British Columbia, Vancouver, BC, Canada; ^5^ Department of Microbiology and Immunology, University of British Columbia, Vancouver, BC, Canada; ^6^ Department of Medical Genetics, University of British Columbia, Vancouver, BC, Canada; ^7^ Biotechnology - Biomedical Science and Technology (BST), University of Applied Sciences, Mannheim, Germany; ^8^ Pascal Biosciences, Seattle, WA, United States; ^9^ School of Medicine, Koç University, Istanbul, Türkiye; ^10^ Department of Chemistry, University of British Columbia, Vancouver, BC, Canada; ^11^ Department of Zoology, University of British Columbia, Vancouver, BC, Canada; ^12^ Department of Urological Science, University of British Columbia, Vancouver, BC, Canada

**Keywords:** cannabinoids, major histocompatibility class I, MHC, cytolytic T lymphocyte, CTL, immune escape, immune edited, metastatic cancers

## Abstract

Emerging cancers are sculpted by neo-Darwinian selection for superior growth and survival but minimal immunogenicity; consequently, metastatic cancers often evolve common genetic and epigenetic signatures to elude immune surveillance. Immune subversion by metastatic tumours can be achieved through several mechanisms; one of the most frequently observed involves the loss of expression or mutation of genes composing the MHC-I antigen presentation machinery (APM) that yields tumours invisible to Cytotoxic T lymphocytes, the key component of the adaptive cellular immune response. Fascinating ethnographic and experimental findings indicate that cannabinoids inhibit the growth and progression of several categories of cancer; however, the mechanisms underlying these observations remain clouded in uncertainty. Here, we screened a library of cannabinoid compounds and found molecular selectivity amongst specific cannabinoids, where related molecules such as Δ9-tetrahydrocannabinol, cannabidiol, and cannabigerol can reverse the metastatic immune escape phenotype *in vitro* by inducing MHC-I cell surface expression in a wide variety of metastatic tumours that subsequently sensitizing tumours to T lymphocyte recognition. Remarkably, H3K27Ac ChIPseq analysis established that cannabigerol and gamma interferon induce overlapping epigenetic signatures and key gene pathways in metastatic tumours related to cellular senescence, as well as APM genes involved in revealing metastatic tumours to the adaptive immune response. Overall, the data suggest that specific cannabinoids may have utility in cancer immunotherapy regimens by overcoming immune escape and augmenting cancer immune surveillance in metastatic disease. Finally, the fundamental discovery of the ability of cannabinoids to alter epigenetic programs may help elucidate many of the pleiotropic medicinal effects of cannabinoids on human physiology.

## Introduction

The ability of the adaptive immune system to seek and destroy emerging tumours is reliant upon immune surveillance by cytolytic T lymphocytes (CTLs) (1). In perhaps one of the most fascinating molecular mechanisms in all biology, CTL recognize major histocompatibility class I (MHC-I) molecules that act as peptide receptors that have been loaded with small fragments of proteolytically generated foreign peptides, through a process termed antigen processing (1). As a result of immunological tolerance mechanisms, CTL generally ignore healthy cells that display MHC-I loaded with self-peptides and rather focus on cells expressing MHC-I bound foreign antigens such as viral peptides or abnormal peptides, including cancer antigens.

During the evolution of cancers, genetic and epigenetic alterations occur that enable the cancers to become metastatic (2) and are referred to as a metastatic signature. A common form of metastatic signature is one that allows the cancer to evade the immune system. In the context of CTL recognition of MHC-I peptide complexes, there are various mechanisms acting exclusively or in concert, that underpin escape from immune surveillance. These include the absence or low expression of MHC-I molecules due mutations or epigenetic regulation, tumour-induced T-lymphocyte anergy, and/or defects in MHC-I antigen presentation machinery (APM) (3, 4).

MHC-I molecules are required for antigen presentation to CTLs, and the regulation of natural killer cells. Thus, alteration in the expression of surface MHC-I has been determined as an important tumour escape mechanism (1). Under the negative selection of CTLs, this immune escape (1) (also termed immune-edited) phenotype can even reach a penetrance of 100% in some carcinoma types (5, 6). Since entry of processed peptides into the endoplasmic reticulum (ER) *via* transporters associated with antigen processing 1 and 2 (TAP-1/2) is required for the assembly of MHC-I peptide complexes, the loss of TAP-1/2 greatly contributes to a functional defect in the antigen processing and presentation pathway (1). These phenotypic changes that appear at the clonal level are associated with malignant transformation (7–9) and allow malignant cells to evade immune surveillance by ultimately disabling the cells’ ability to present cell surface peptides. Tumour cells that have defects in the APM have a selective advantage compared to other tumour cells that retain a functional APM, conferring on them a greater metastatic potential. Several types of cancer, including breast cancer (10, 11), renal cell carcinoma (12), melanoma (13, 14), colorectal carcinoma (15), head and neck squamous cell carcinoma (16), cervical cancer (17), and finally prostate carcinoma show a clear correlation between MHC-I down-regulation and poor prognosis (18–20). The increasing frequency of immune escape tumour variants in many forms of metastatic cancers is a predictor of disease progression as well as predictor of poor patient outcome. Relatively few attempts have been made to treat metastatic disease by directly trying to overcome the APM deficits in immune escape tumour variants as a therapeutic modality. During our earlier studies, we revised the conclusions of Stutman (21), and formally demonstrated, for the first time to our knowledge, that T-lymphocytes are indeed required for cancer immune surveillance *in vivo* (1). Specifically, animals genetically lacking T-lymphocytes lose the ability to survey and resist tumour expansion of even MHC-I expressing tumours. Furthermore, we demonstrated that functional expression of APM components in tumours is required to enable immune surveillance and the loss of APM components that often occurs in metastatic tumours, allowing them to grow and expand even in wild-type animals possessing a normal T-lymphocyte compartment (1). We subsequently elaborated on this point by directly restoring APM expression *in vivo* using viral vectors that introduced the missing APM into tumours in animals with ongoing metastatic disease, resulting in a dramatic reduction of tumour growth. Thus, we recognized that the possibility to restore CTL recognition of metastatic carcinomas by complementation and replacing missing APM components may have a clinical application in cancer immunotherapies (1, 22–27). Intriguingly, APM deficiency is not exclusively regulated by defects or mutations in the APM genes, but it may be epigenetically regulated as well (28) and can be restored by treatment with histone deacetylase inhibitors (HDACi) such as trichostatin-A (TSA) (28, 29) or complementation with cytokines such as IL-33 (30, 31) or interferon gamma (IFN-γ) (1, 2, 22–27).

Based on these observations, we focused on the discovery of natural small molecules that may reverse immune escape and thereby improve tumour antigen recognition by the immune system with the goal of enhancing protective immune responses. Among the compounds promoting immune recognition that we identified were cannabinoids. For decades, cannabinoids have been reported to specifically inhibit cancer growth, but the mechanism remains undescribed (32). However, perhaps slowing the pace of experimentally documenting the exact mechanism underlying their medicinal effect, cannabinoids are actually a diverse class of compounds that act on the brain and other tissues of the body by targeting cannabinoid receptors, other G protein coupled receptors, ion channels, transporters, as well as enzymes (33). The phytocannabinoids, for example, are found in *Cannabis sativa* and other plants, and many of these natural products have demonstrated pharmacological properties. Endocannabinoids such as anandamide, on the other hand, are naturally produced in the body, and act as natural ligands for cannabinoid receptors (34). Artificially manufactured synthetic cannabinoids also exhibit activity on cannabinoid receptors, while having structural similarity to naturally occurring cannabinoids. The most controversial form of these is the primary psychoactive compound in *Cannabis*, known as tetrahydrocannabinol (THC). However, it should be emphasized that more than 100 different cannabinoids have been isolated from *Cannabis*. Some cannabinoids have agonist activity while others have antagonist activity on the characterized cannabinoid receptors. Consequently, mixtures of cannabinoids, such as those found in *Cannabis* or in crude extracts of *Cannabis*, are likely to have contradictory activities that likely continues to obscure their true clinical potential. Furthermore, such natural preparations are notoriously difficult to reproducibly manufacture resulting in differences from batch to batch. Therefore, it may be advantageous for a purified or synthetic cannabinoid or cannabinoid derivative to be advanced for clinical development.

We have developed a method to indirectly screen for compounds that increase MHC-I expression in metastatic tumours. This approach has identified numerous cannabinoid compounds that increased MHC-I expression and promoted immune recognition of metastatic cancer cells.

## Materials and methods

### Cell culture

The murine lung carcinoma cell line, TC-1, was derived from primary lung epithelial cells of a C57BL/6 mouse that were immortalized using the amphotropic retrovirus vector LXSN16 carrying the Human Papillomavirus E6/E7 oncogenes and subsequently transformed with pVEJB plasmid expressing the activated human H-Ras oncogene (35). The metastatic cell line, A9, is a derivative of TC-1 that was generated *in vivo* after an immunization strategy in animals bearing the original TC-1 parental cells to drive selection for clones with enhanced immunoresistance (36, 37). In contrast to the parental TC1 cells, which display high expression of TAP-1 and MHC-I, A9 cells express nearly undetectable levels of MHC-I. Both of the aforementioned cell lines were cultured in Dulbecco’s modified Eagle’s medium (Gibco) containing 10% fetal bovine serum (FBS, Gibco), 100 U/mL penicillin-streptomycin (Gibco) and incubated at 37°C in a 5% CO_2_ humidified atmosphere. Other cell lines including murine 4T1, CT26, EMT6, Renca, B16-410, LLC, MC38, B16F10 and A20 and human COLO 205, SK-N-MC, SNU-C1, DLD-1, LS123, LS411N, LoVo, SK-MEL-2, NCI-H146, A431 and SK-MEL-2 were cultured as described above.

### Mice

OT1 mice (Strain #:003831, The Jackson Laboratory) contain transgenic inserts for mouse Tcra-V2 and Tcrb-V5 genes. The transgenic T cell receptor was designed to recognize ovalbumin peptide residues 257-264 (OVA257-264) in the context of H2Kb (CD8 co-receptor interaction with MHC class I) and this results in the development of MHC class I-restricted, ovalbumin-specific, CD8+ T lymphocytes (OT-I T lymphocytes).

### Flow cytometry

Cell lines were trypsinized (0.05%; Gibco), washed twice with PBS (Gibco), and stained with allophycocyanin (APC)-conjugated anti-mouse H-2K^b^ antibody (1:200; Biolegend) suspended in 150 μL of FACS buffer (PBS + 2% FBS) for 20 minutes at 4°C. Cells were washed with PBS twice and then resuspended in 200 μL FACs buffer containing 1 μL of 7-aminoactinomycin D (7AAD) viability stain (Biolegend). Flow cytometry was performed on a LSRII (BD Biosciences) and analysis was done using FlowJo software (BD; flow cytometry analysis software, version 6).

A flow cytometry assay was also developed for testing the MHC-I-inducing activity of IFN-γ and cannabinoids in human and mouse cancer cell lines. All cells were obtained from ATCC and were cultured as recommended by the supplier. Cells were seeded at subconfluent densities in 96-well flat bottom plates and, after 24 hours of incubation at 37°C, 5% CO_2_ in a humidified chamber, culture wells were supplemented with test concentrations of cannabinoids, recombinant human or mouse IFN-γ (positive control, R&D Systems), or vehicle control (1% DMSO in culture medium). The treated plates were then incubated another 48 hr, after which cells were collected by centrifugation in the case of non-adherent cells, and treatment with TrypLE Express (ThermoFisher Scientific) in the case of adherent cells. Harvested cells were washed thrice with cold FACS buffer (1% BSA in PBS) and stained for one hr on ice. All human cell lines were stained with a 1:20 dilution of the FITC-conjugated, mouse anti-human pan-HLA W6/32 antibody (Life Technologies). Mouse cell lines 4T1, CT26, EMT6, Renca, and A20 were stained with 2.5 μg/ml of the FITC-conjugated mouse anti-mouse H-2K^d^/H-2D^d^ MHC-I allotype antibody (clone 34-1-2S, BioLegend), while mouse cell lines B16-410, LLC, MC38, and A9 were stained with 10 μg/ml of the FITC-conjugated mouse anti-mouse H-2K^b^/H-2D^b^ MHC-I allotype antibody (clone 28-8-6, BioLegend). After washing the cells in FACS buffer, stained cells were quantitated by flow cytometry using a FACSCalibur (BD Biosciences), after which data were analyzed with FlowJo software. Viability was determined using the vital dye, SYTOX Red (ThermoFisher Scientific).

A variety of cannabinoids, including endo-, phyto-, and synthetic cannabinoids, all acquired from Cayman Chemical (Ann Arbor, Michigan, USA), were tested in the COLO 205 MHC-I induction assay. The 371 synthetic cannabinoids were arrayed as 10 mM stocks in DMSO in a 96-well plate screening format (Cayman #9002891). The endocannabinoids were provided as concentrated stocks in ethanol (AEA) or acetonitrile (2-AG), and the phytocannabinoids provided as either concentrated stocks in methanol or as dry powder which was reconstituted in methanol. All cannabinoid stocks were diluted with positive displacement pipettors under appropriate safety constraints into culture medium for assay of MHC-I induction activity. All Schedule I regulated cannabinoids were handled in a DEA-certified laboratory (USA) or PHAC-certified laboratory (Canada) with special exemption status. Cannabigerol (CBG) was stored as a stock solution in DMSO.

### Proxy cytolytic T lymphocyte assay

1x10^6^ A9 cells were plated onto a 6 well plate in two mL of RPMI medium (Advanced RPMI-1640 Medium; # 12633020, Gibco), 100 U/mL of Penicillin-Streptomycin (P+S) (#15070063, Thermo Fisher), 1% L-Glutamine (#25030081, Gibco), and 10% FBS. A9 cells were treated with 55 μM (8.8 ng/ml) CBG, 167 μM (18.6 ng/ml) CCP, 5.9 nM (100 ng/ml) IFN-γ, or 1% DMSO vehicle. After 24 hours at 37°C, 5% CO_2_, the ovalbumin peptide, SIINFEKL (#257-264, Genscript), was added to the A9 cells. Following an additional incubation for 24 hours, CD8^+^ T cells were collected from OT1 mouse spleens. Spleens were minced and passed through a 100-micron cell strainer (#352260, Falcon). Red Blood Cell ACK lysis buffer (#A10492-01, Gibco) was used to remove red blood cells from the spleen isolate. CD8^+^ Untouched Mouse CD8^+^ lymphocytes (Dynabeads, #11417D, ThermoFisher Scientific) was used to enrich CD8^+^ T lymphocytes, as per the manufacture’s protocol. The medium was removed from the A9 cells and they were washed three times with PBS before fresh RPMI was added to the wells. An extra well of untreated A9 cells was counted and used as a baseline count. CD8^+^ T cells were counted and afterwards treated with carboxyfluorescein succinimidyl ester (CFSE; #79898, Biolegend) per the manufacturer’s protocol, before being co-cultured with A9 cells at a 1:1 or a 1:5 ratio of T lymphocytes tumour cells. For the positive control, T cells were stimulated 24 hours later using CD28 monoclonal antibody clone 37.51 (#14-0281-86, eBioscience) at a concentration of 5 μg/mL and CD3e monoclonal antibody clone 145-2C11 at 10 μg/mL, (eBioscience, #14-0031-86). CD8^+^ T lymphocytes and A9 cells were harvested for analysis in flow cytometry. CD8^+^ T lymphocytes were stained with CD8 PE-efluor 610 antibody (#60-0081-82, Invitrogen). A9 Cells were stained using PE H-2KB antibody (#12-5958-82, Invitrogen), and 7AAD viability dye (#420404, Biolegend) in FACS buffer. All mouse experiments were approved by the Animal Care Committee at UBC. Animals were maintained and euthanized under humane conditions in accordance with the guidelines of the Canadian Council on Animal Care.

### Cytokine secretion profile of A9 cells upon treatment of small molecules

All compounds, (Cannabigerol, IFN gamma) were dissolved in 1% Dimethyl Sulfoxide (DMSO) (Catalog #276855, Sigma) in media (1%DMSO). 1x10^6^ A9 cells were plated onto a 6 well plate in two mL of DMEM media. Twenty-four hours after seeding, cells were cultured at the optimum concentrations of Cannabigerol 0.055 μmol, or 5.832x10^-6^ nmol IFN gamma or 1% DMSO vehicle for 48 hours. Relative expression levels of 111 soluble mouse proteins including cytokines, chemokines and growth factors were evaluated using the Proteome Profiler Mouse XL cytokine array kit (R&D System, ARY028) following the manufacturer’s instructions. Spot densities on the array film were detected and quantified using Image J analysis software Image J protein analyzer add-on on a scanned version of the film. Quantification of the spot intensity in the arrays was conducted with background subtraction in ImageJ. To determine fold change, the treatment values of IFN gamma microarrays were divided by the DMSO negative control values of the concurring spots. A value of 1 was subtracted from the absolute value of this fold change, to correct the value of DMSO to “0”. The experiment was done in technical replicates. Key cytokines that showed changes in expression levels were further characterized by pathway analysis for over-represented pathway identification through Reactome Database release 65, Pathway Brower Version 3.5. The results displayed concerning the arraying conducted is an average of an N of 2.

### Reverse transcription and RT-qPCR

RNA was isolated using the RNeasy plus mini kit. RNA was reverse transcribed into cDNA using the superscript II reverse transcription kit (Catalog # 18064014, Invitrogen). Quantitative RT PCR was done using 10nm of primer and 1uL of BioRad SYBR Green master mix (Catalog#1725271, Biorad). RT-qPCR was done on 7500 Fast Real-Time PCR System from Applied Biosystems 40 cycles (95°C denaturing for 15 seconds, 60°C annealing for one minute).

### Bioinformatic analysis of H3K27Ac data

#### Processing of ChIP-seq data

Raw sequencing data was aligned to mouse reference genome (mm10) with BWA mem (v0.7.6a) with option (-M). Peak calling was done with MACS2 (v2.1.2) with FDR cutoff 0.01 and option (-f BAMPE) (38, 39). During peak calling process, input samples were used as a background control.

#### Overlap analysis of H3K27Ac peaks of DMSO, cannabigerol and IFN-gamma samples and Venn diagram

We used Intervene (v0.6.4) to generate a Venn diagram of the comparison with “–save-overlaps” option to obtain sample-specific or common binding regions (40). Comparison of DMSO and IFN-gamma generated the region sets lost and gained which gained represent IFN-gamma specific and lost is DMSO specific. Further, gained and lost regions then intersected with Cannabigerol regions to create genomic region set “gained-Cannabigerol” and “lost-Cannabigerol” respectively.

## Results

### MHC-I downregulation is reversible by IFN-γ in human and murine cancer cell lines

**Figure 1:**
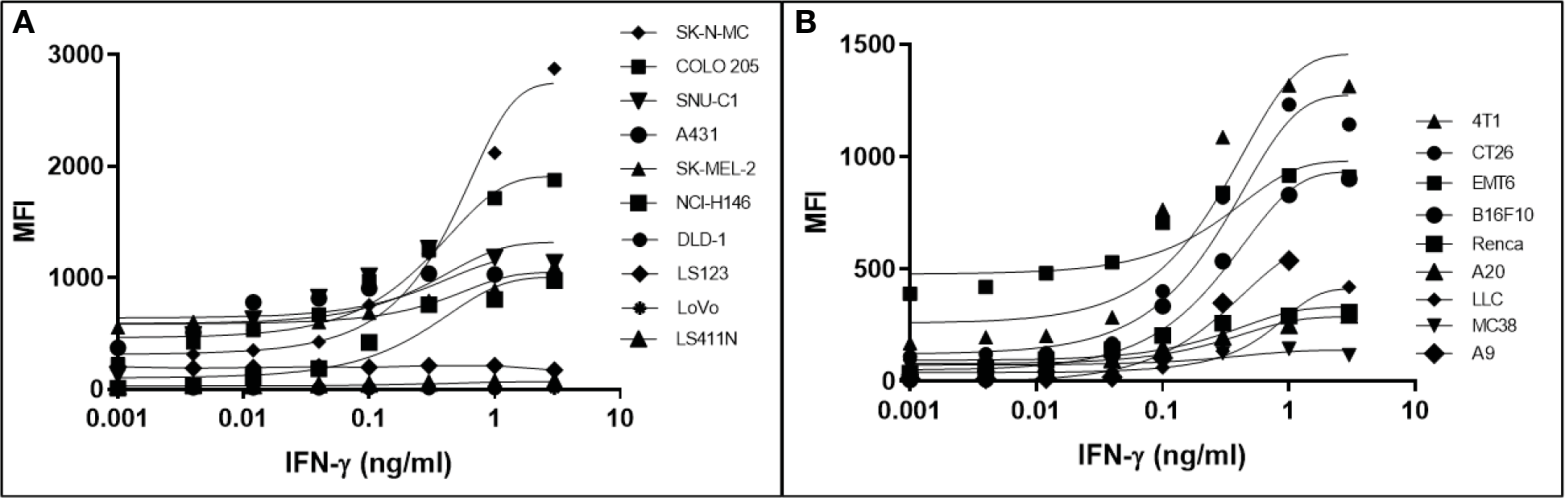
Downregulation of MHC-I expression is reversible by in most human **(A)** and mouse **(B)** cancer cell lines. The MHC-1-low or -null cell lines were treated with a titration of human **(A)** or mouse **(B)** IFN-γ for 48 hr, washed, and MHC-I expression determined by flow cytometry. The pan-human HLA antibody, W6/32, was used to stain the human cell lines, and mouse cell lines were stained with the MHC-I allotype-specific 34-1-2S (4T1, CT26, EMT6, Renca, A20) or 28-8-6 (B16-410, LLC, MC38, A9) antibodies.

It has been determined that only 30%-40% of lost MHC-I expression in tumours is due to a genetic lesion impacting the structural genes involved in APM (28, 41–43), suggesting that MHC-I expression might be restorable in cancers with intact but under-expressed APM genes. To test this possibility, we treated various human and mouse cancer cell lines with dose titrations of recombinant human or mouse IFN-γ, respectively. The cells were incubated with IFN-γ for 48 hrs in a humidified chamber at 37°C, stained with fluorescent haplotype-appropriate MHC-I antibody, then signal was determined by flow cytometry. Cancers represented in this experiment include brain (SK-N-MC), breast (4T1, EMT6), colorectal (COLO 205, SNU-C1, DLD-1, LS123, LS411N, LoVo, CT26, MC38), kidney (Renca), lung (NCI-H146, LLC, A9), lymphoid (A20), and skin (A431, SK-MEL-2, B16F10). IFN-γ induced MHC-I expression to varying degrees in a dose-dependent manner in 6/10 (60%) human (**Figure 1A**) and 8/9 (89%) mouse cell lines (**Figure 1B**). A9 was treated with a maximum dose of mouse IFN-γ of 1ng/ml. This was based on a previous titration and time course with IFN-γ to establish maximal induction. These numbers are consistent with the previously reported values (41, 44) and indicate that, while cell surface MHC-I may be downregulated, it can be induced in many human and murine cancers.

### Cannabigerol induces MHC-I expression in mouse and human metastatic carcinomas

**Figure 2:**
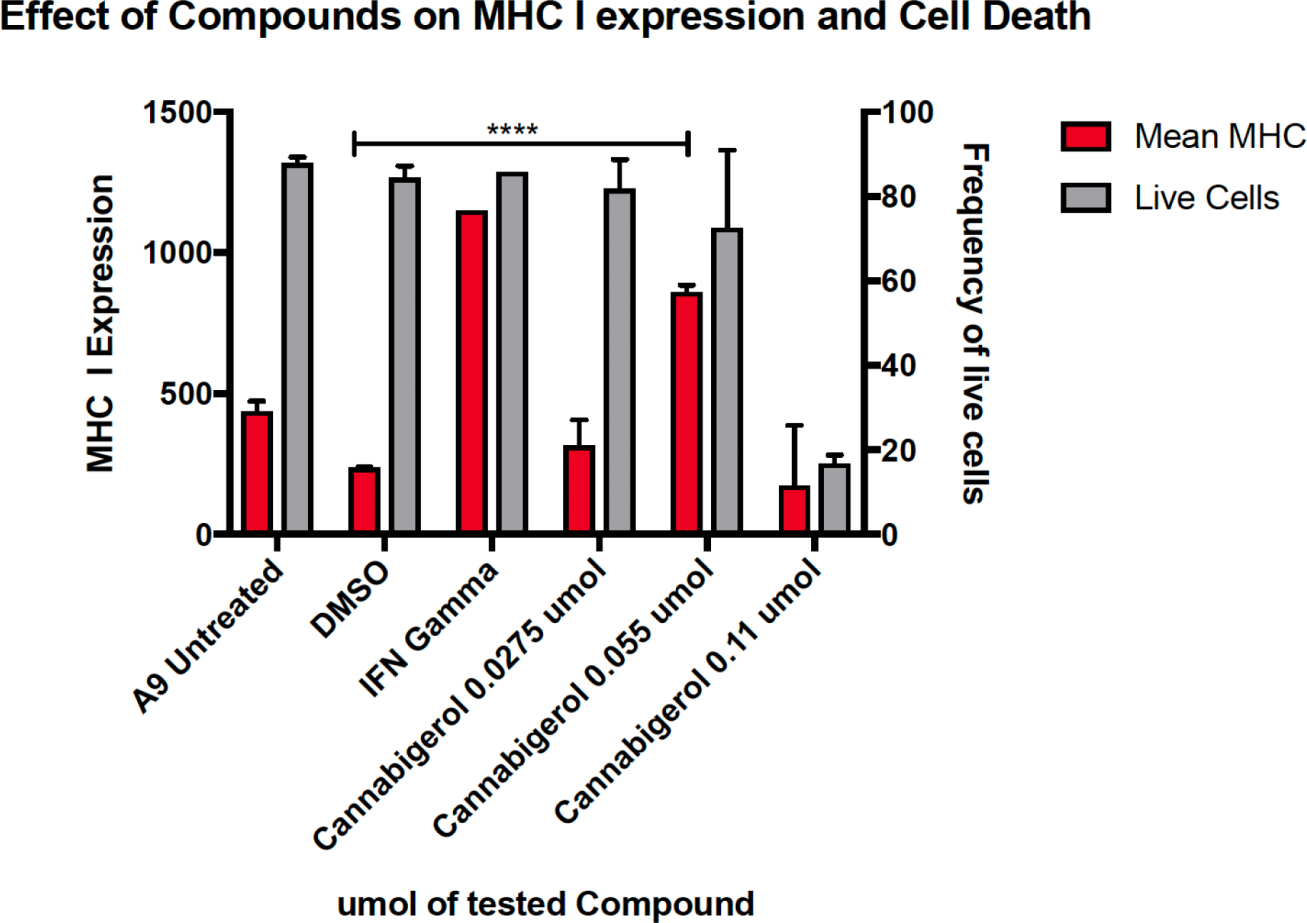
MHC-I downregulation is reversible by IFN-γ or cannabigerol in metastatic murine cells. The murine lung cancer cell line, A9 was treated with various doses of CBG and compared with induction by IFN-γ. MHC-I was measured by flow cytometry after 48 hr. In comparison to the vehicle control DMSO, significant induction (*) was demonstrated at 0.0275 µM and 0.055 µM of CBG (red) while cell viability (grey) was maintained above 70% for both concentrations of CBG. These data are representative of three independent experiments.

**Figure 3:**
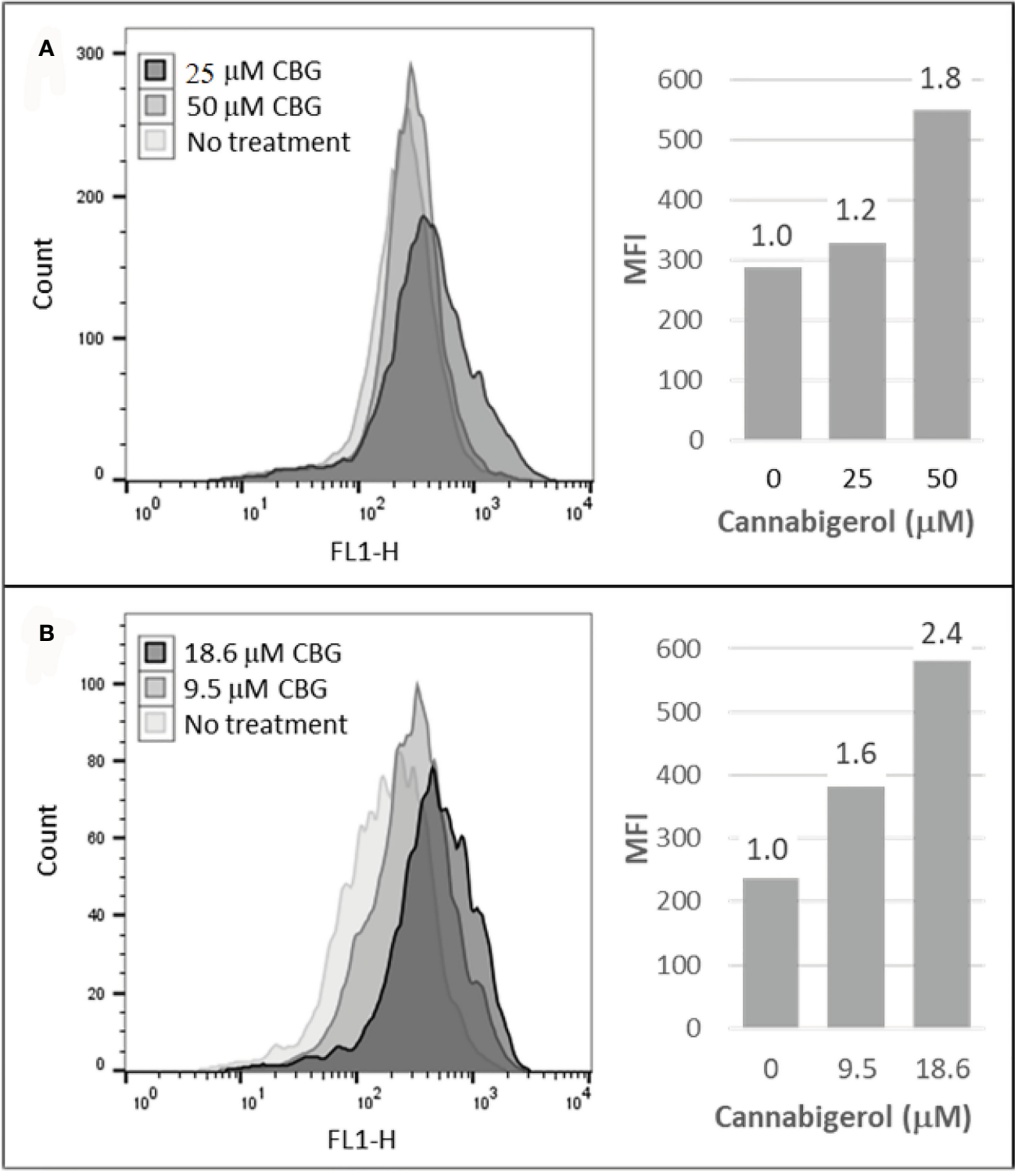
Cannabigerol induces MHC-I expression in human and mouse metastatic carcinomas. **(A)** The human colorectal cancer cell line, COLO 205 was treated with 25 and 50 μM CBG and **(B)** the mouse breast cancer cell line 4T1 was treated with 9.5 and 18.6 μM CBG. In both experiments, MHC-I was measured by flow cytometry after 48 hr. Left panel: flow cytometry histogram. Right panel: mean fluorescent intensities (MFI) for each treatment condition**;** fold increase in MHC-I expression (treatment MFI/no treatment MFI) is indicated above each bar. These data are representative of three independent experiments.

The high percentage of cancer cell lines with low but inducible APM (**Figure 1**) prompted us to examine if cannabinoids can also induce MHC-I in metastatic cells (45). The effect of the cannabinoid, cannabigerol was examined and compared with the effect of IFN-γ on the metastatic murine lung carcinomas, A9 cell line. MHC-I protein upregulation in the presence of cannabigerol was nearly as great as with IFN-γ at a concentration of 0.055 μmol. This was determined using FACS and was statistically significant, with a p-value of less than 0.0001 between DMSO treated cells and cannabigerol treated metastatic cells at a concentration of 0.055 μmol while using an ordinary one-way ANOVA. (**Figure 2**). We next examined if the induction of MHC-I in response to cannabigerol could be generalizable to human metastatic carcinomas and other murine metastatic carcinomas. **Figure 3** illustrates the response of the cells after 48 hrs of treatment with a suboptimal and an optimal dose of the cannabinoid. The human colorectal cancer cell line COLO 205 was treated with 25 and 50 μM CBG (**Figure 3A**), and the mouse breast cancer cell line 4T1 was treated with 9.5 and 18.6 μM CBG (**Figure 3B**). Both cell lines respond with MHC-I upregulation in a dose-dependent manner. With ascending dosing, COLO 205 responded with 1.2- and 1.8-fold, and 4T1 displayed 1.6- and 2.4-fold increase in MHC-I levels. Shifts in MHC-I expression as those observed in (**Figure 3**) have been demonstrated to functionally reconstitute CTL recognition in a previous study conducted by Jefferies et al. (46), indicating that as few as 10 MHC-I peptide complexes are able to be recognized by CTL. As illustrated in the flow cytometry histograms, most if not all, cells in the population responded to cannabigerol treatment, driving a rightward shift of the peak.

### Phytocannabinoids as a class induce MHC-I expression

**Figure 4:**
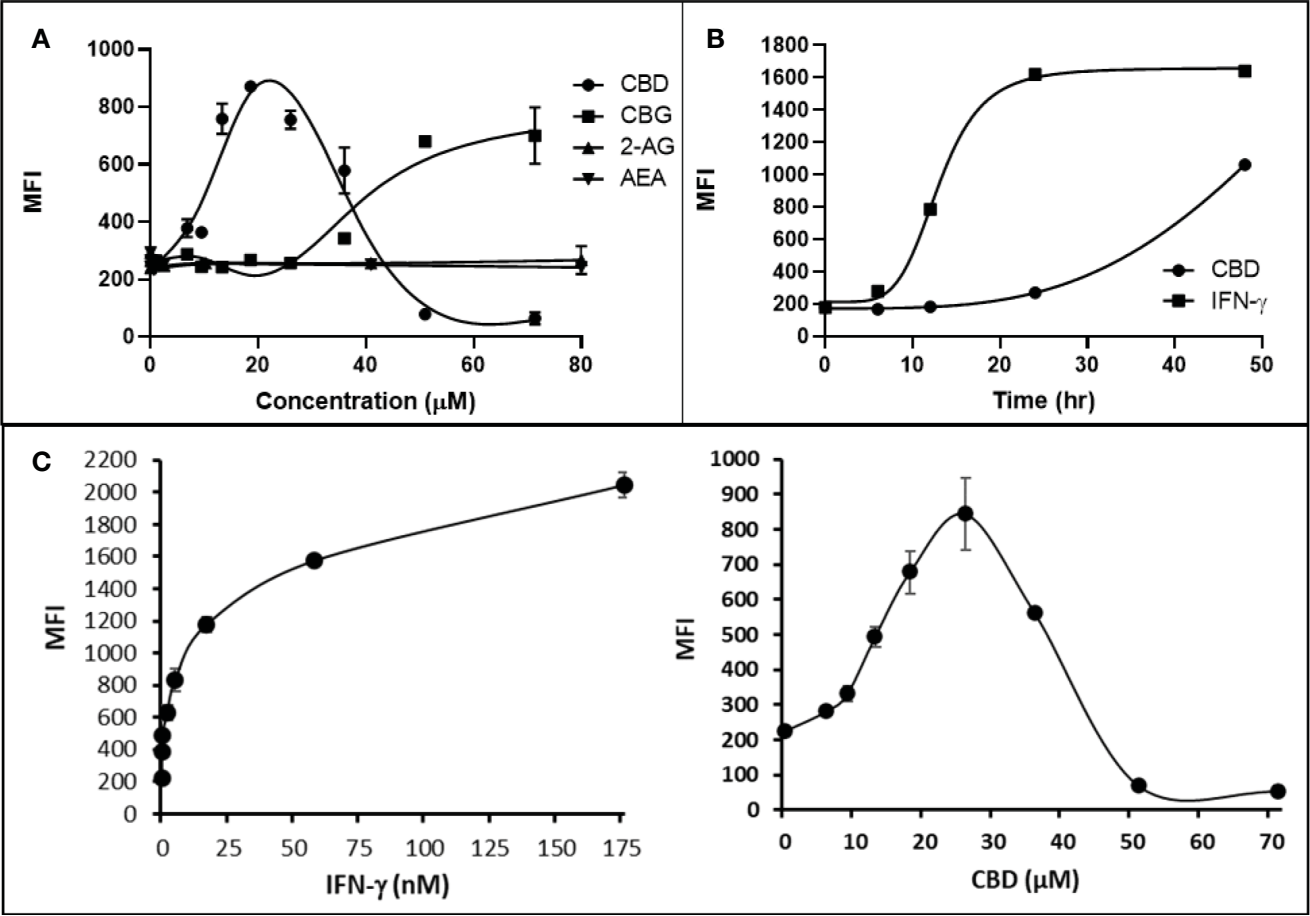
Phytocannabinoids, but not endocannabinoids, induce MHC-I expression in a dose- and time-dependent manner. **(A)** The endocannabinoids 2-AG and AEA had no effect on MHC-I expression in COLO 205 cells as determined by flow cytometry, in contrast to the activity of the phytocannabinoids CBD and CBG after 48 hr of treatment. **(B)** An MHC-I induction time course shows that elevated MHC-I is first detectable at 24 hr in cultured COLO 205 cells treated with 15 µM CBD but has reached maximum expression by 24 hr in cells treated with 3 ng/ml IFN-γ. **(C)**. Parallel dose titrations spotlight the dramatic impact of IFN-γ and CBD on MHC-I expression in COLO 205 cells. The titrations also clarify differences in potency and dose effect between the cytokine and the cannabinoid. The dose titrations were conducted in triplicate in the same experiment and are representative of numerous IFN-γ and CBD comparisons. Statistical analysis using a two-sided T-test with unequal variances revealed that every titration data point has a p-value of less than 0.005 vs. the untreated sample. MFI: mean fluorescent intensity.

Cannabigerol is only one of many cannabinoid-like molecules with potential biopharmaceutical activity. Because of its relatively modest potency in the MHC-I induction assay compared to IFN-γ (see **Figures 1**, **3**), we set out to test additional cannabinoids. Phytocannabinoids are derived from certain plants, most notably *Cannabis sativa*, and endocannabinoids are naturally present in vertebrates. Both classes exert their physiological effects *via* the various receptors and pathways discussed above. We tested the two best characterized endocannabinoids, 2-arachidonoylglycerol (2-AG) and N-arachidonoylethanolamine (AEA, anandamide) along with a second phytocannabinoid, cannabidiol (CBD) for MHC-I-inducing activity. The endocannabinoids did not induce MHC-I expression above baseline in COLO 205 cells at any of the concentrations tested (**Figure 4A**), suggesting these molecules do not play a physiological role in regulating MHC-I. However, along with CBG, CBD was able to induce MHC-I expression by these cells, with EC_50_ values of 40.3 and 11.1 μM, respectively, in this experiment (**Figure 4A**). The bell shape of the CBD induction curve was unexpected but may relate to the health of the cells exposed to the higher CBD concentrations. We found that overall viability of the cell population decreased at the higher CBD levels and, while dead cells were gated out of the flow cytometry analysis, MHC-I induction may be compromised in the remaining viable cells. Supporting this possibility is the observation that pharmacological induction of ER stress reduced MHC-I gene product expression in a human airway epithelium cell line (47).

Comparison of the kinetics of MHC-I induction suggests that the mechanism by which CBD induces MHC-I in these cells is distinct from that invoked by IFN-γ (see **Figure 1A**). The first detectable increase of expression was at 24 hr in cells treated with CBD (15 μM), and MHC-I levels continued to rise at 48 hr (**Figure 4B**). In contrast, induction by IFN-γ (3 ng/ml) reached a maximum by 24 hr in the same experiment (**Figure 4B**). Further, parallel comparison of the expression of MHC-1 based on dose-dependent titrations of IFN-γ and cannabigerol in COLO 205 cells was able to demonstrate that cannabigerol is approximately 50% as potent as interferon-γ (**Figure 4C**).

The striking differences in MHC-I-inducing activity between the endocannabinoids and the phytocannabinoids are reflected in their structures (see **Table 1**). The functional phytocannabinoids share structural similarities, as do the non-functional endocannabinoids, but the two classes of cannabinoids are structurally quite dissimilar. To determine if MHC-I induction is a feature common to phytocannabinoids as a chemical class, we tested 13 additional phytocannabinoids in the assay (**Table 2**). A nine-point, 1.4-fold dose titration ranging from 100 μM down to 6.8 μM was applied to each cannabinoid. Except for cannabicitran, all of the phytocannabinoids induced MHC-I expression in COLO 205 cells, with EC_50_ values ranging from 11 to >72 μM. Induction levels of two- to three-fold were typical, although four- to six-fold induction was noted for Δ8- and Δ9-tetrahydrocannabinol, cannabivarin, cannabidivarin, and cannabicyclol. Each cannabinoid, except for cannabicitran and cannabigerorcinic acid, caused cell death within the mid to upper range of the titration curve, as reflected in the LC_50_ values. Of all the phytocannabinoids tested, CBD displayed the highest selectivity index (5.4), reflecting the broadest window between biological activity (EC_50_) and toxicity (LC_50_).

**Table 1:** Representatives of cannabinoid classes tested for induction of MHC-I expression in COLO 205 cells.

Cannabinoid Class	Compound	Structure	MHC-I Induction
Endocannabinoid	Arachidonoyl ethanolamide (AEA) 2-Arachidonoyl Glycerol (2-AG)	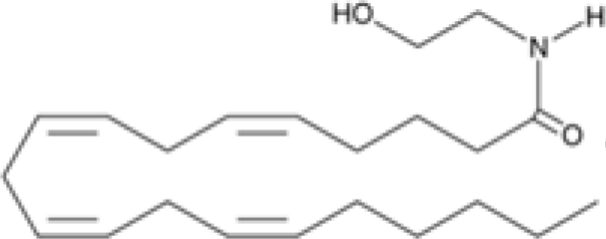 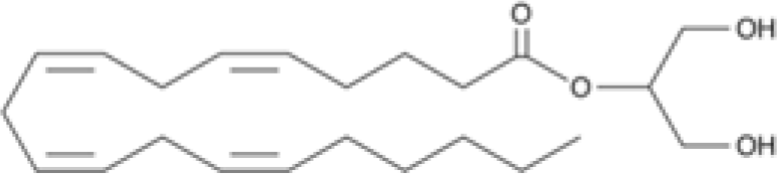	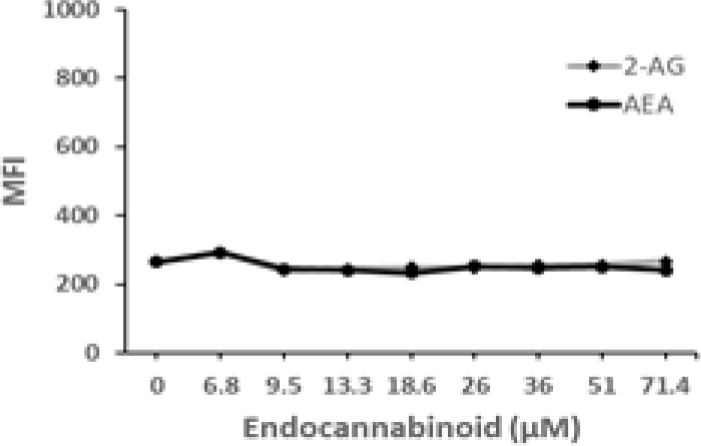
Phytocannabinoid	Cannabigerol (CBG)	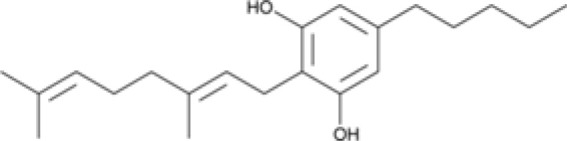	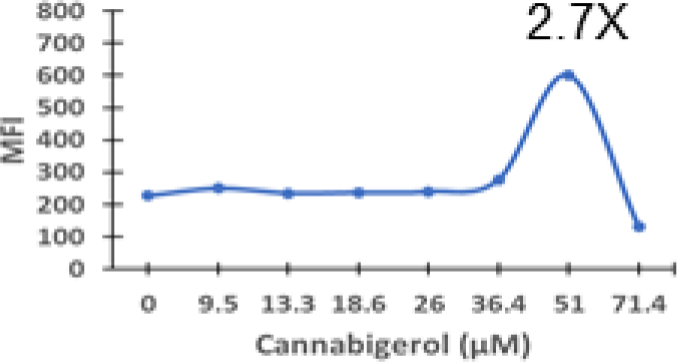
Phytocannabinoid	Cannabidiol (CBD)	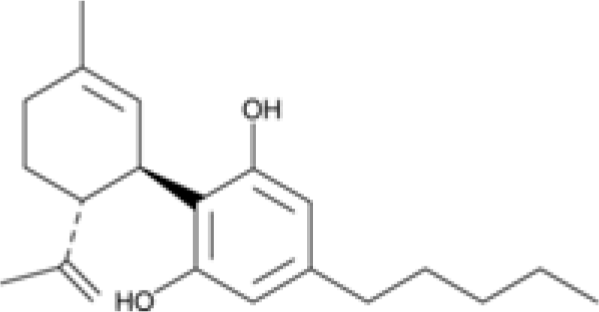	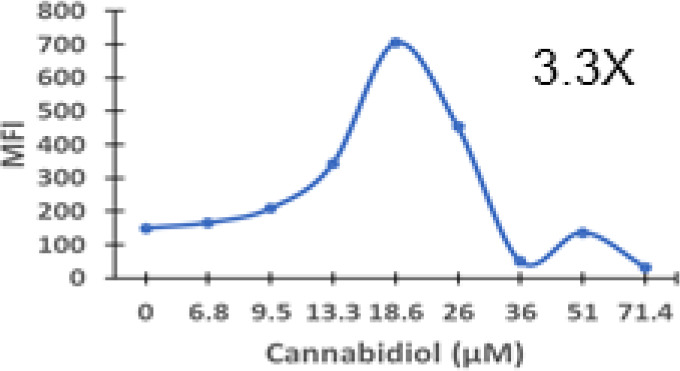
Synthetic cannabinoid, Family 1	JWH 073 2-methyl naphthyl analog	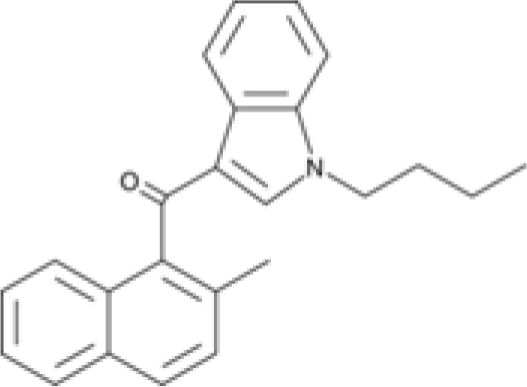	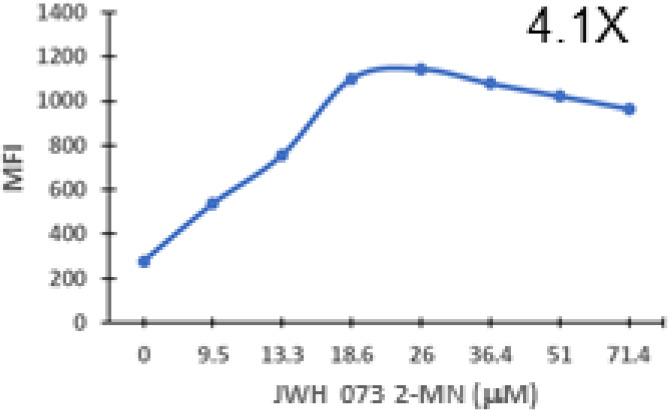
Synthetic cannabinoid, Family 2	5-fluoro PB-22 4-hydroxy-isoquinoline isomer	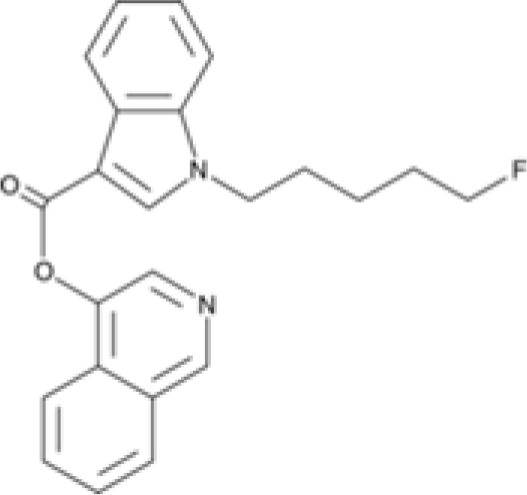	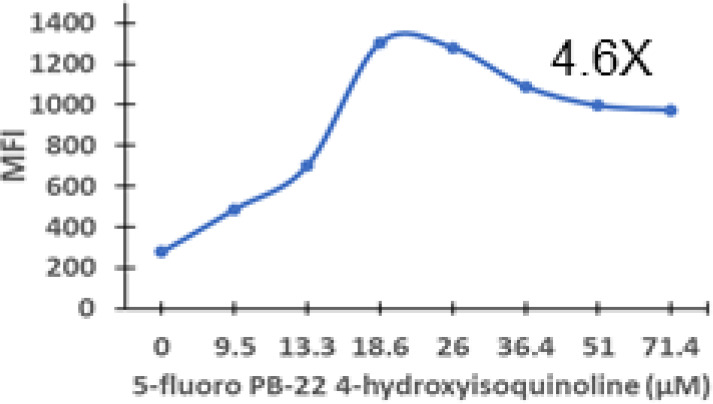
Synthetic cannabinoid, Family 3	MDMB-CHMICA	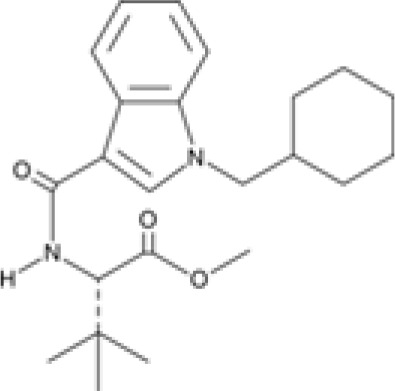	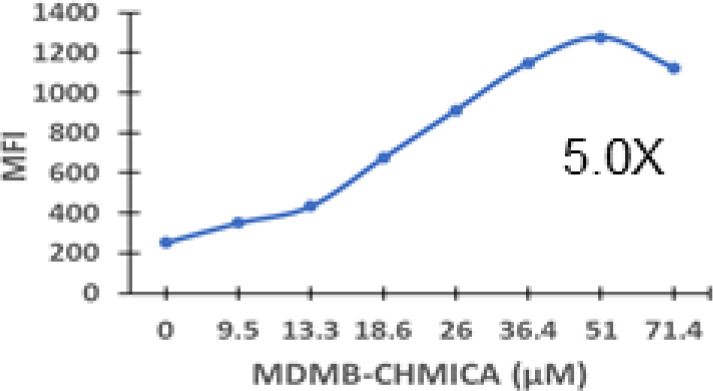
Synthetic cannabinoid, Family 4	(R)-AM1241	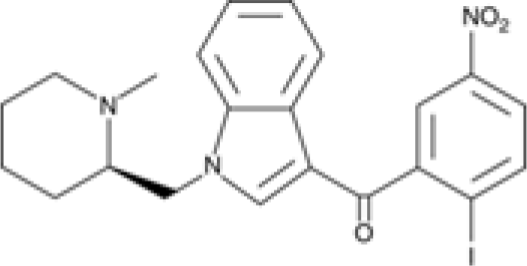	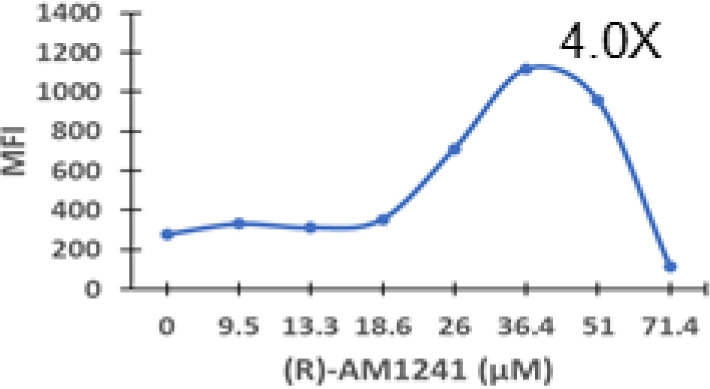
Synthetic cannabinoid, Family 5	UR-144 N-(3-chloropentyl) analog	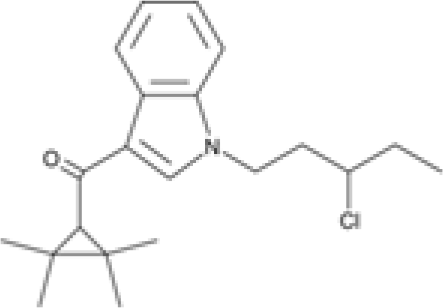	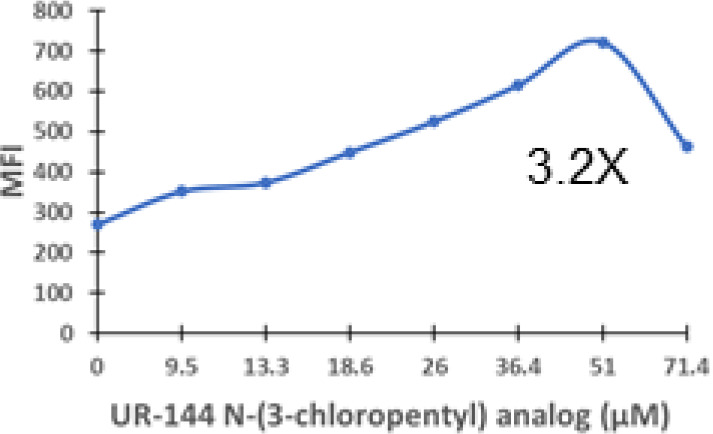
Synthetic cannabinoid, Family 6	URB447	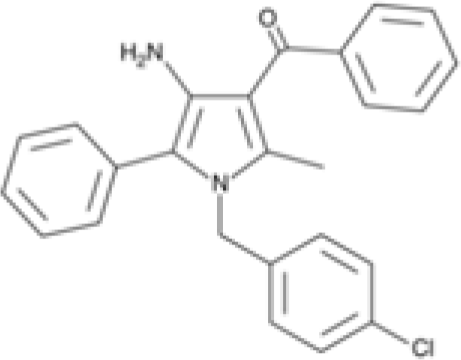	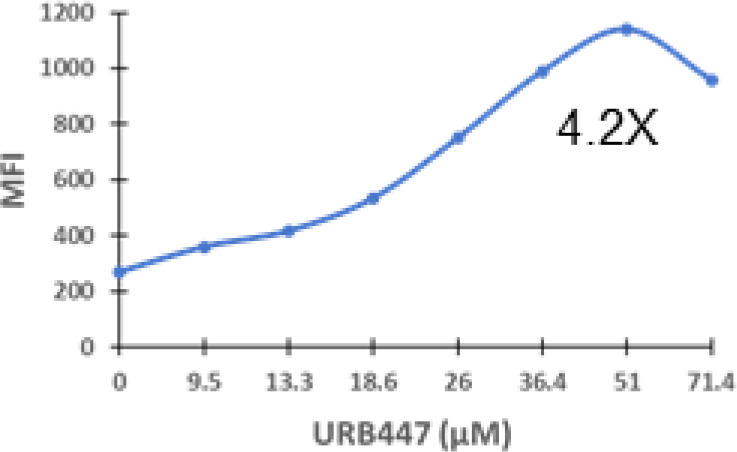
Synthetic cannabinoid, Family 7	(+)-CP 47,497	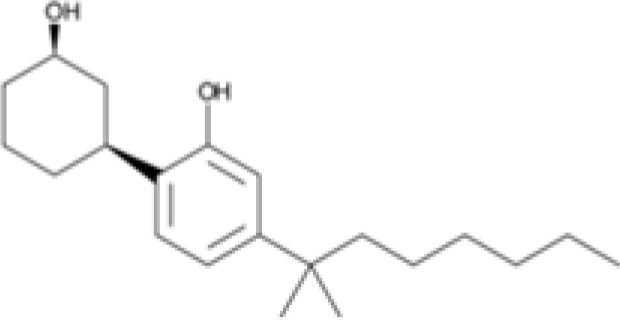	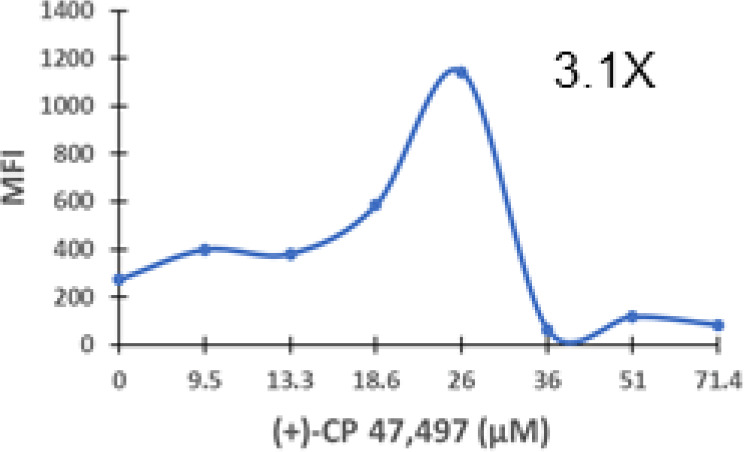

**Table 2:** MHC-1 induction in COLO-205 cells by phytocannabinoids.

Cannabinoid	Fold MHC-I Induction	EC_50_ (µM)	LC_50_ (µM)	Selectivity Index	Structure
Δ 9-Tetrahydrocannabinol	6.1	27.9	55.7	2	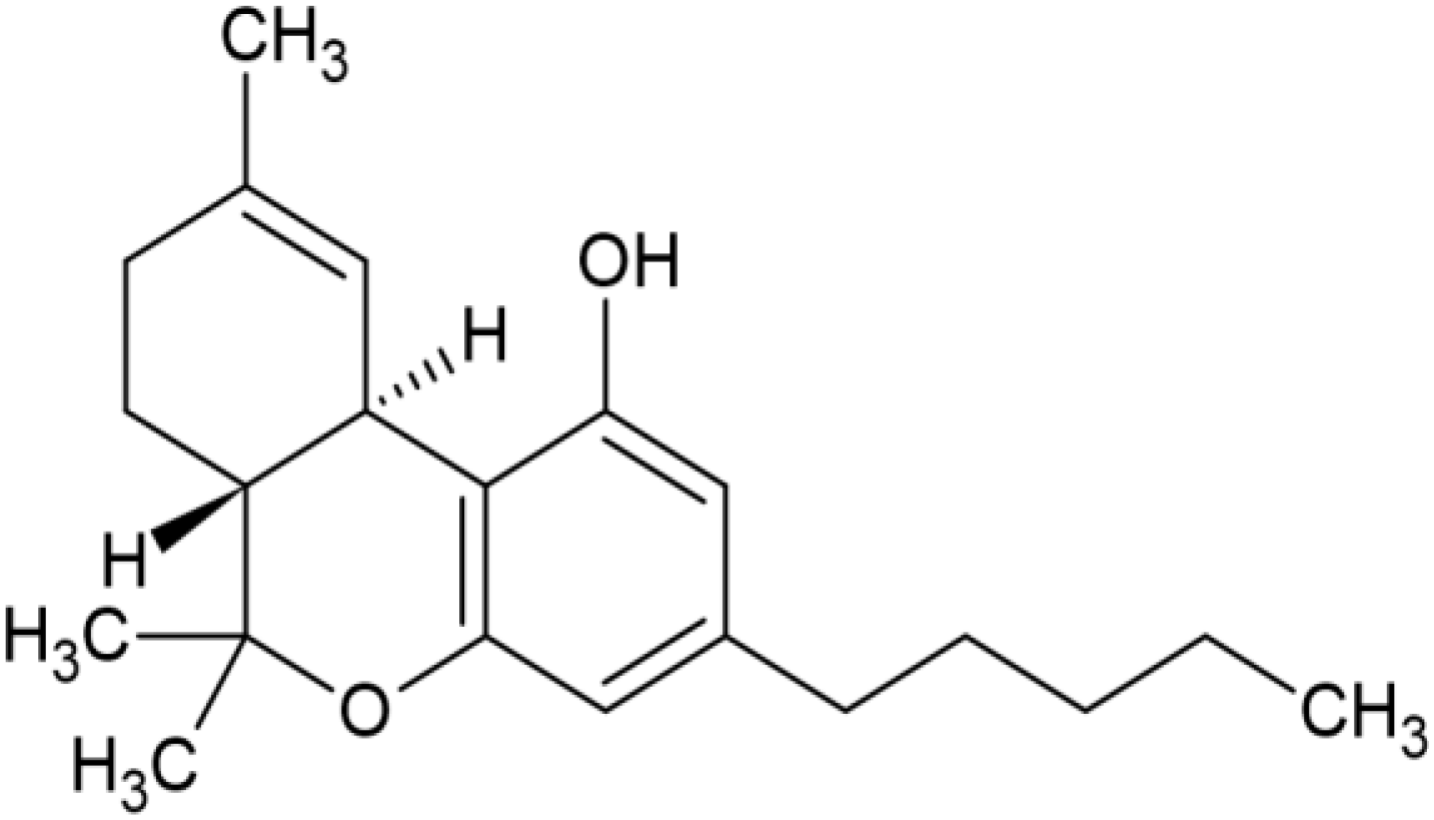
Cannabidivarin	5.5	19.9	35.5	1.8	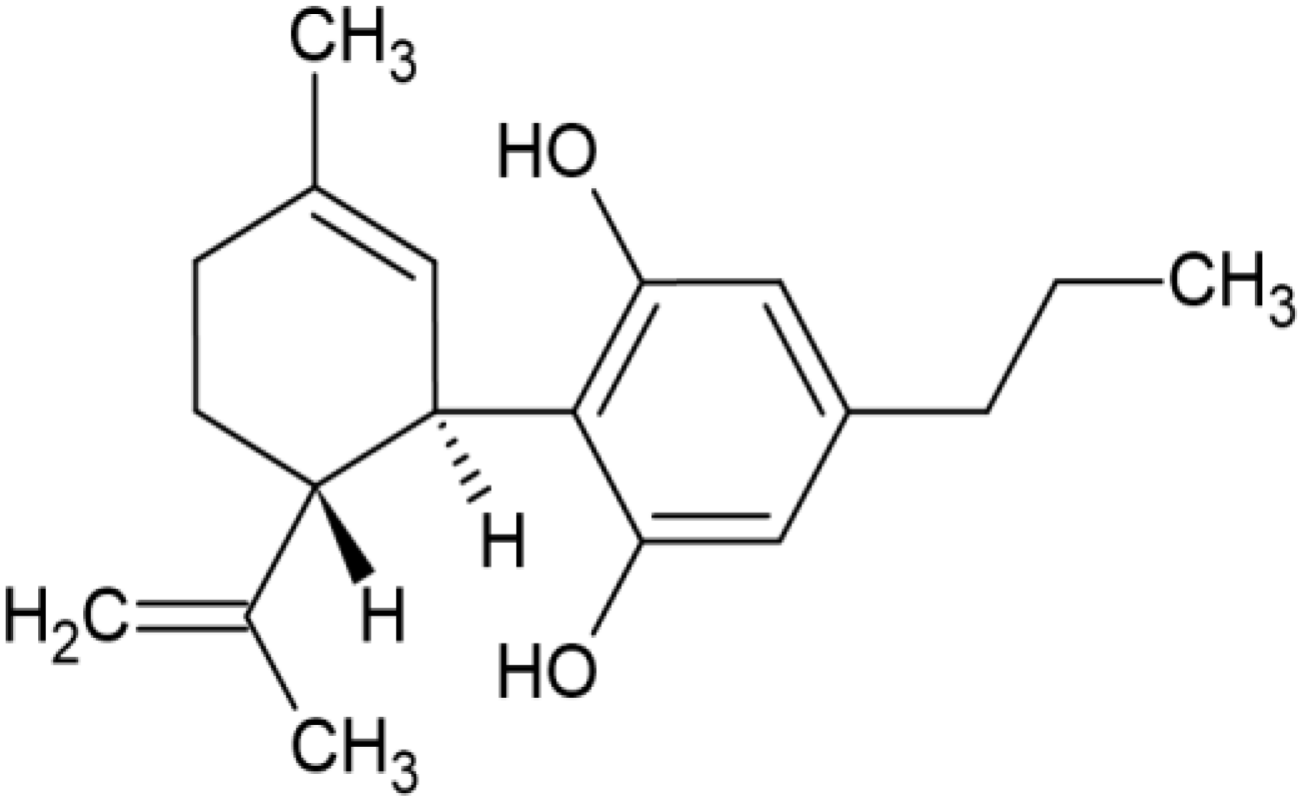
Cannabicyclol	4.2	19.8	26.6	1.3	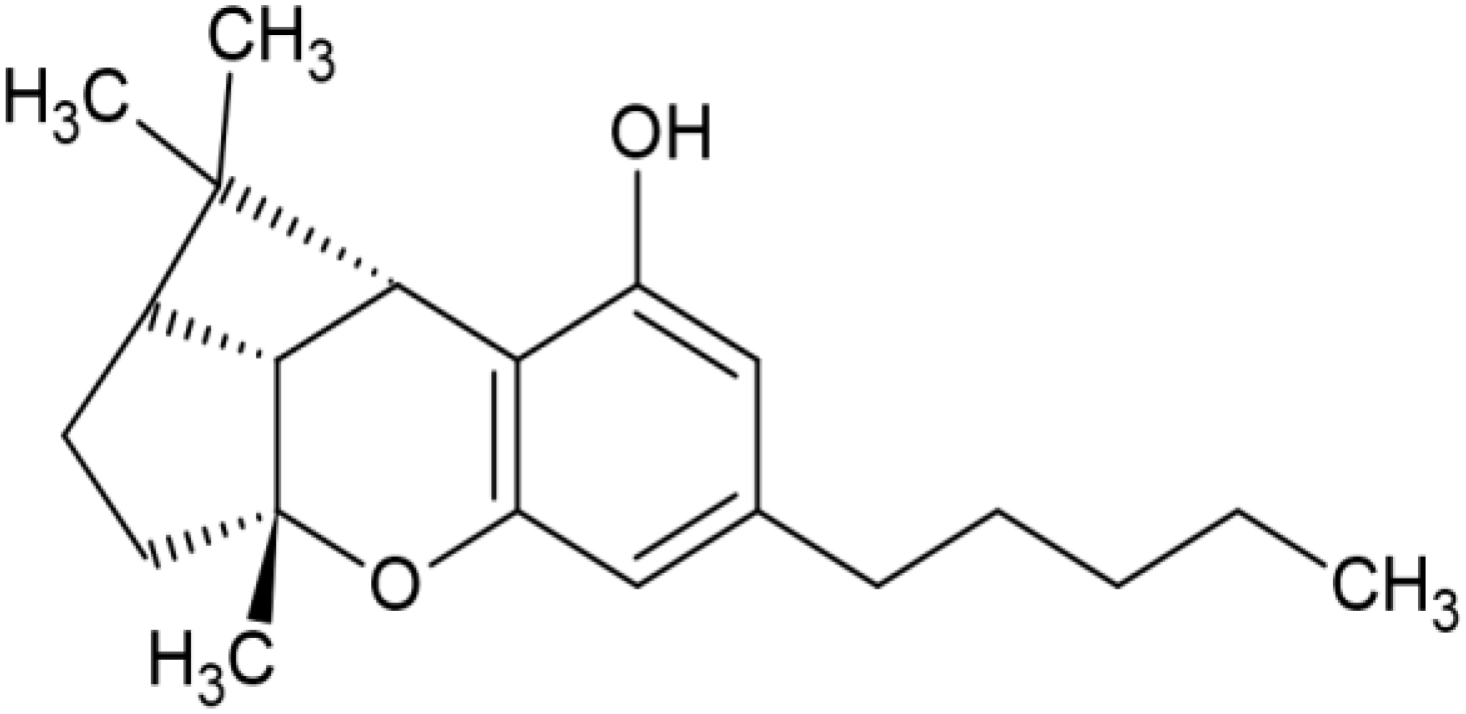
Δ8-Tetrahydrocannabinol	4.1	27.5	57.3	2.1	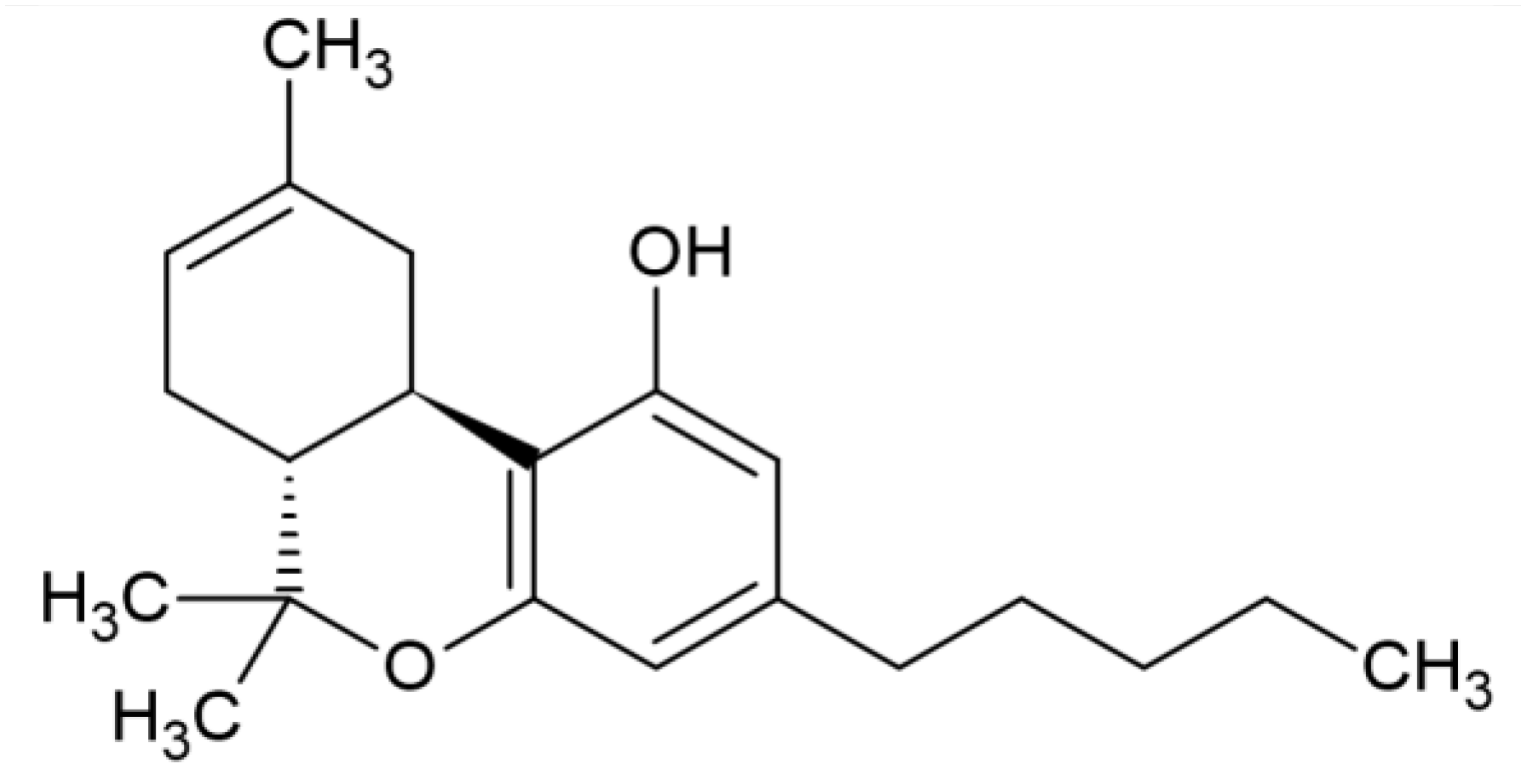
Cannabivarin	3.9	19.9	28.7	1.4	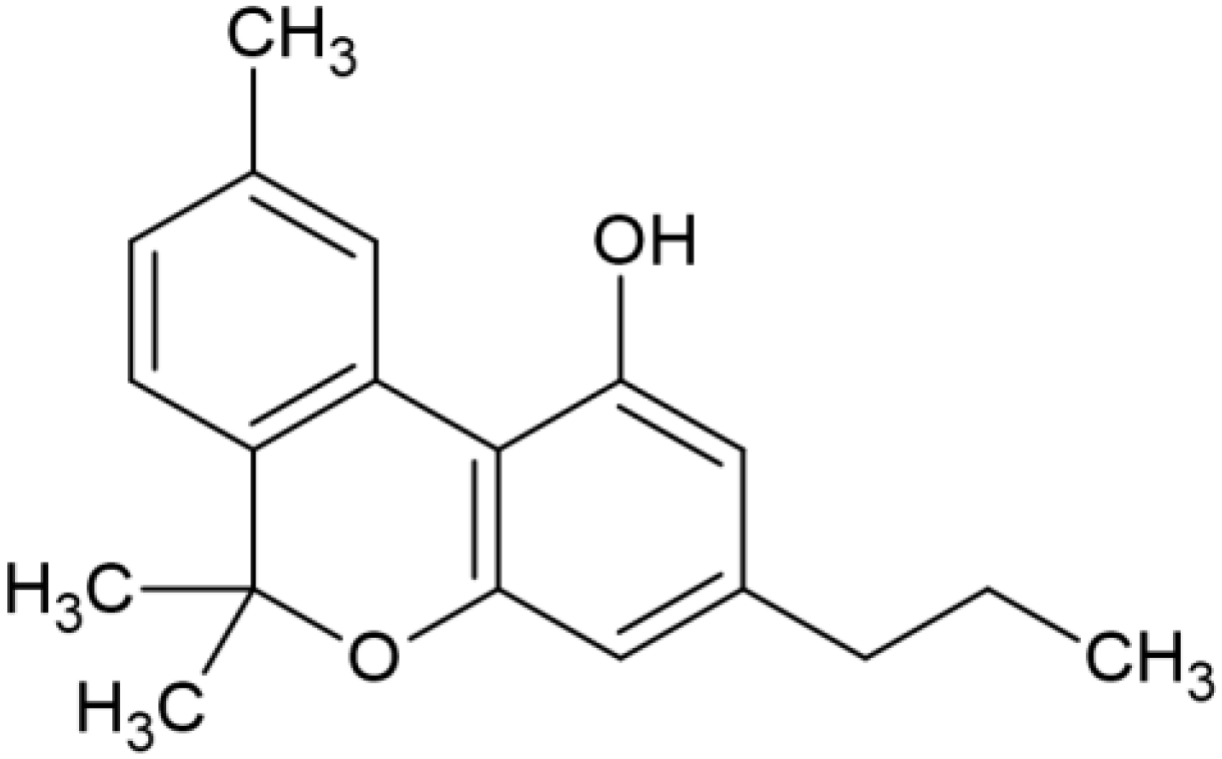
Cannabidiol	3.3	11.1	60.1	5.4	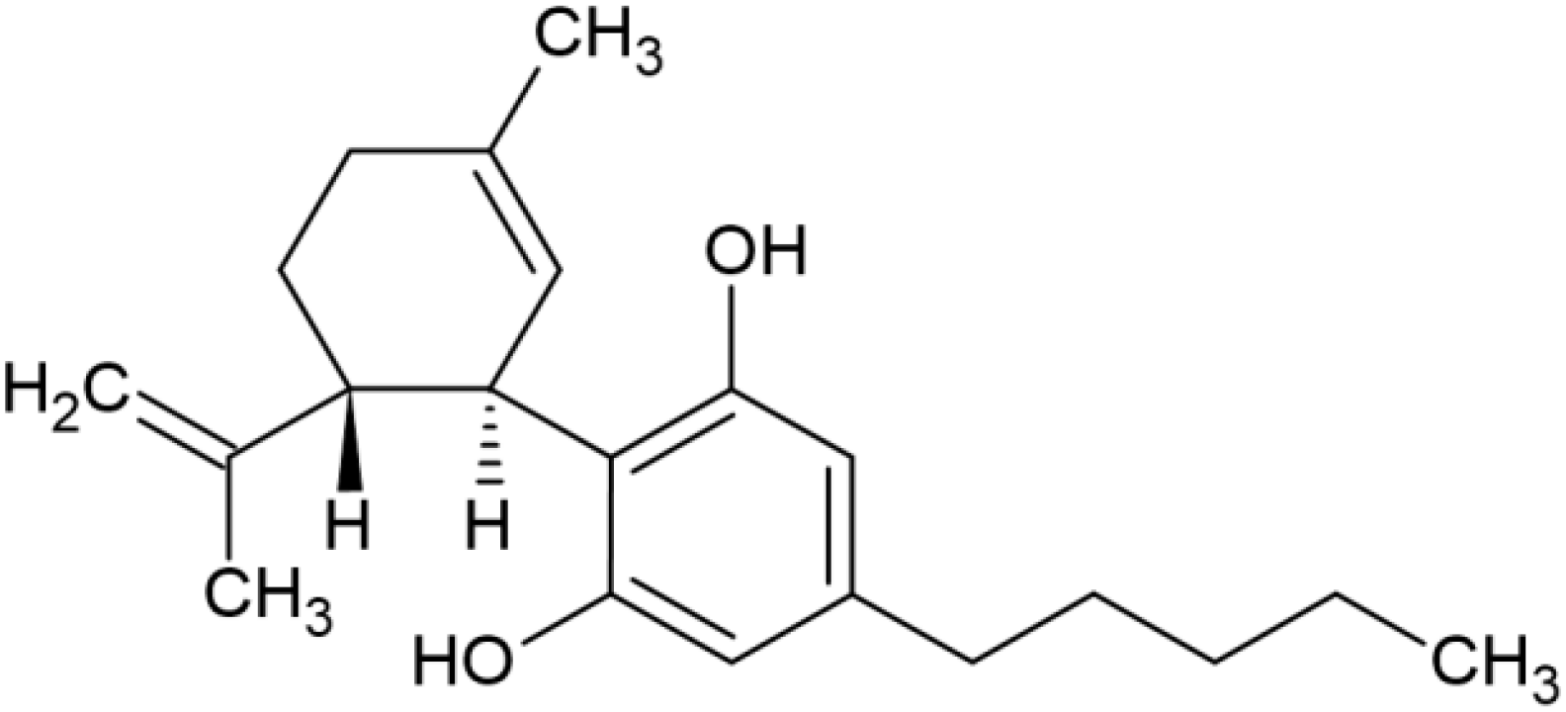
Cannabichromevarin	3.3	32.6	49.5	1.5	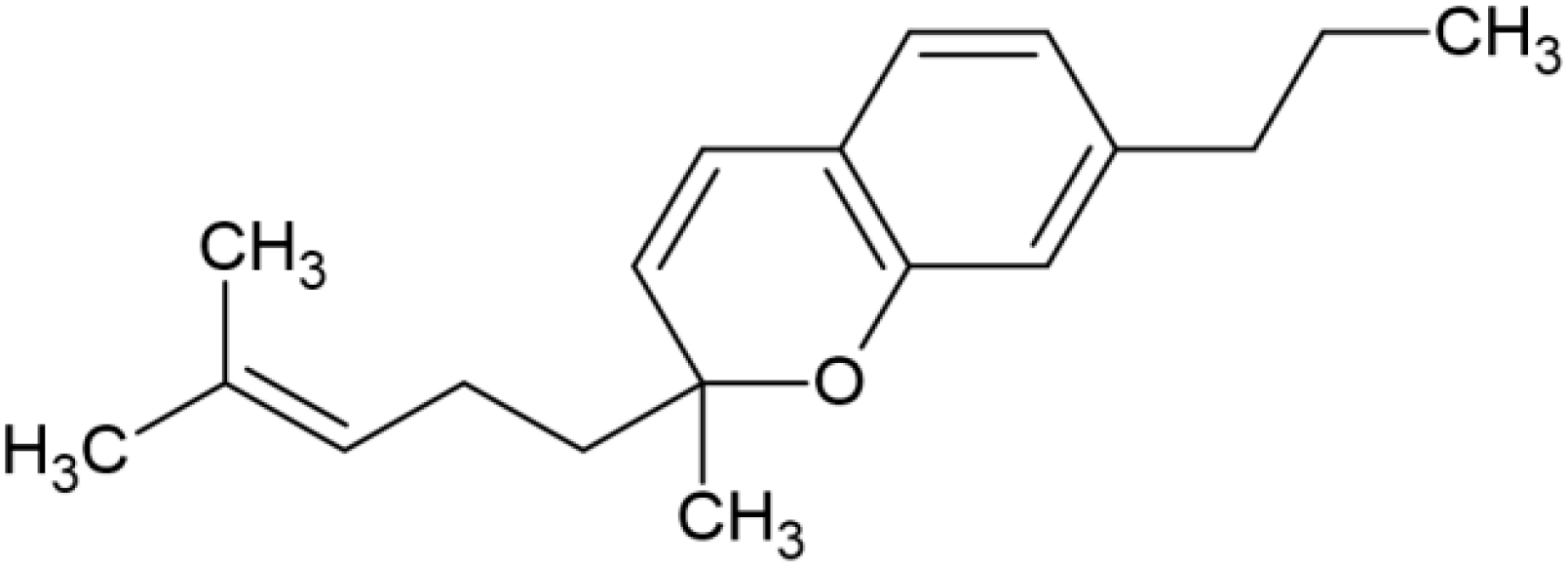
Tetrahydrocannabivarin	3	13.6	35	2.6	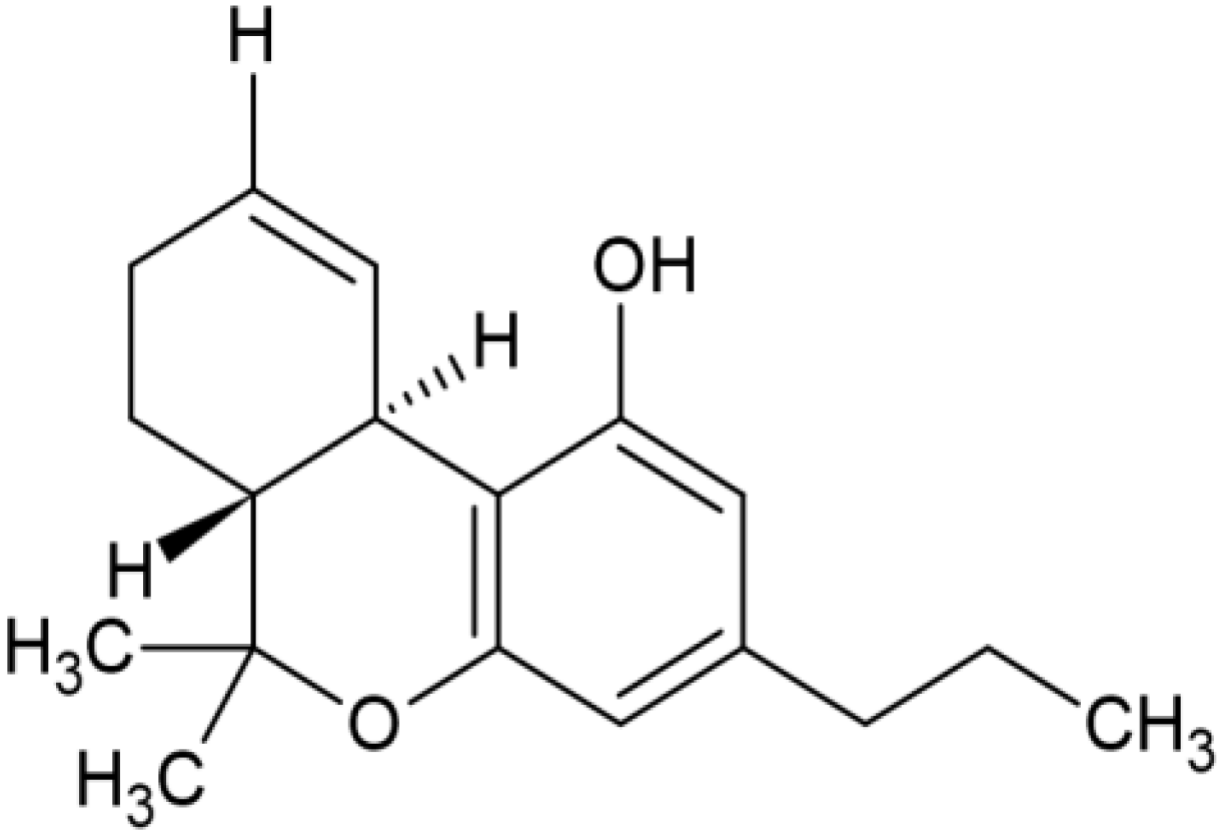
Cannabigerol	2.7	38.9	60.7	1.6	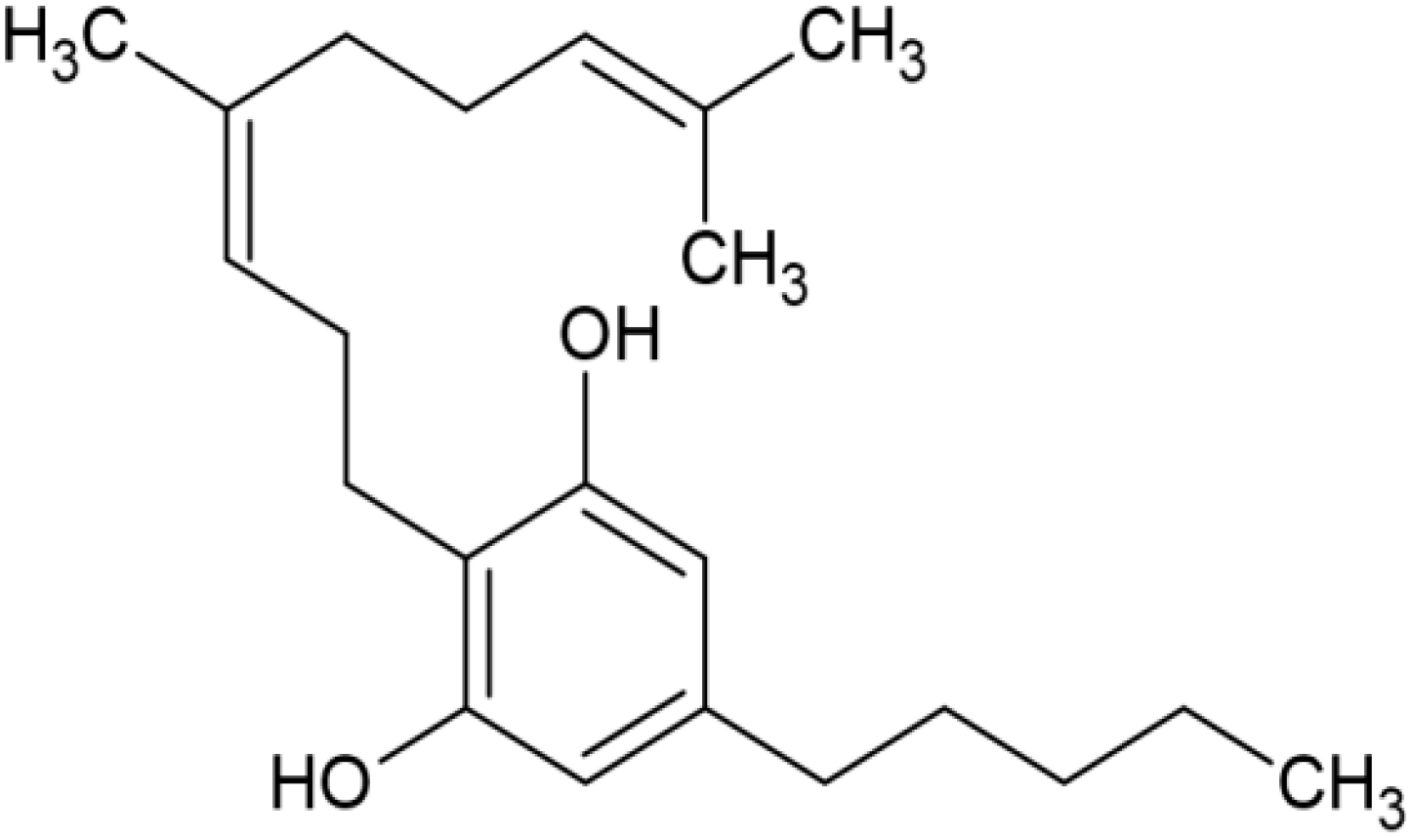
Cannabigerorcin	2.7	37.1	57.6	1.6	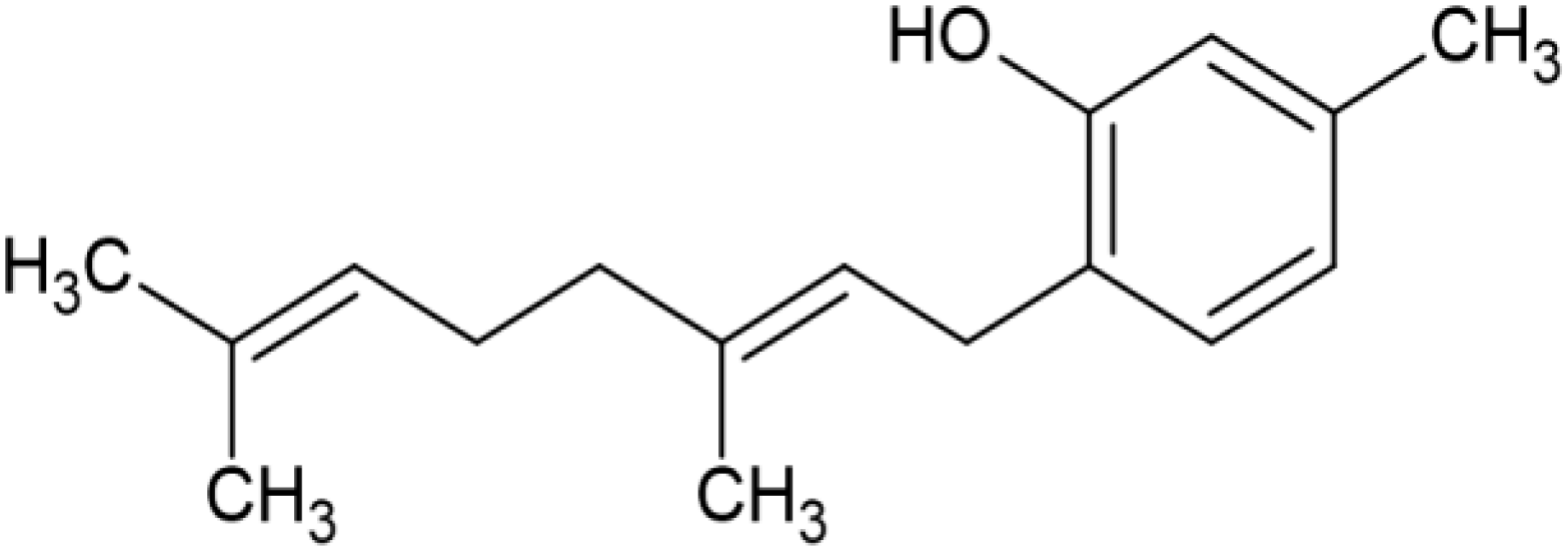
Cannabigerorcinic acid	2.6	>72	NT	ND	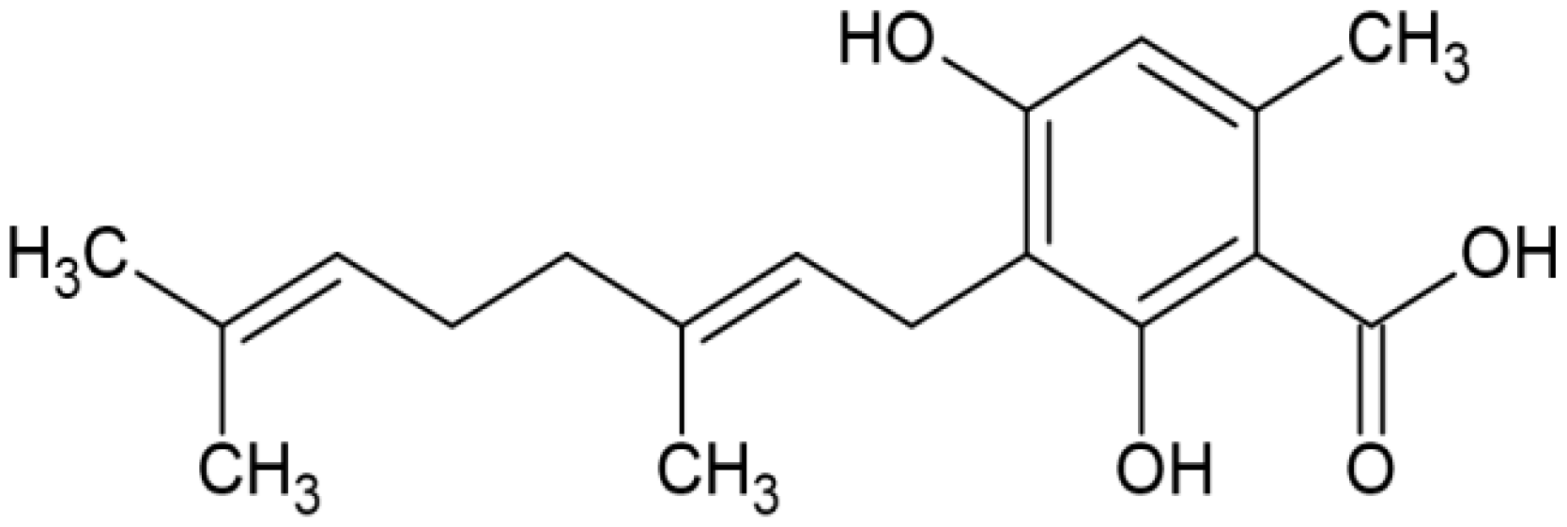
Cannabinol Monomethyl Ether	2.3	40.1	NT	ND	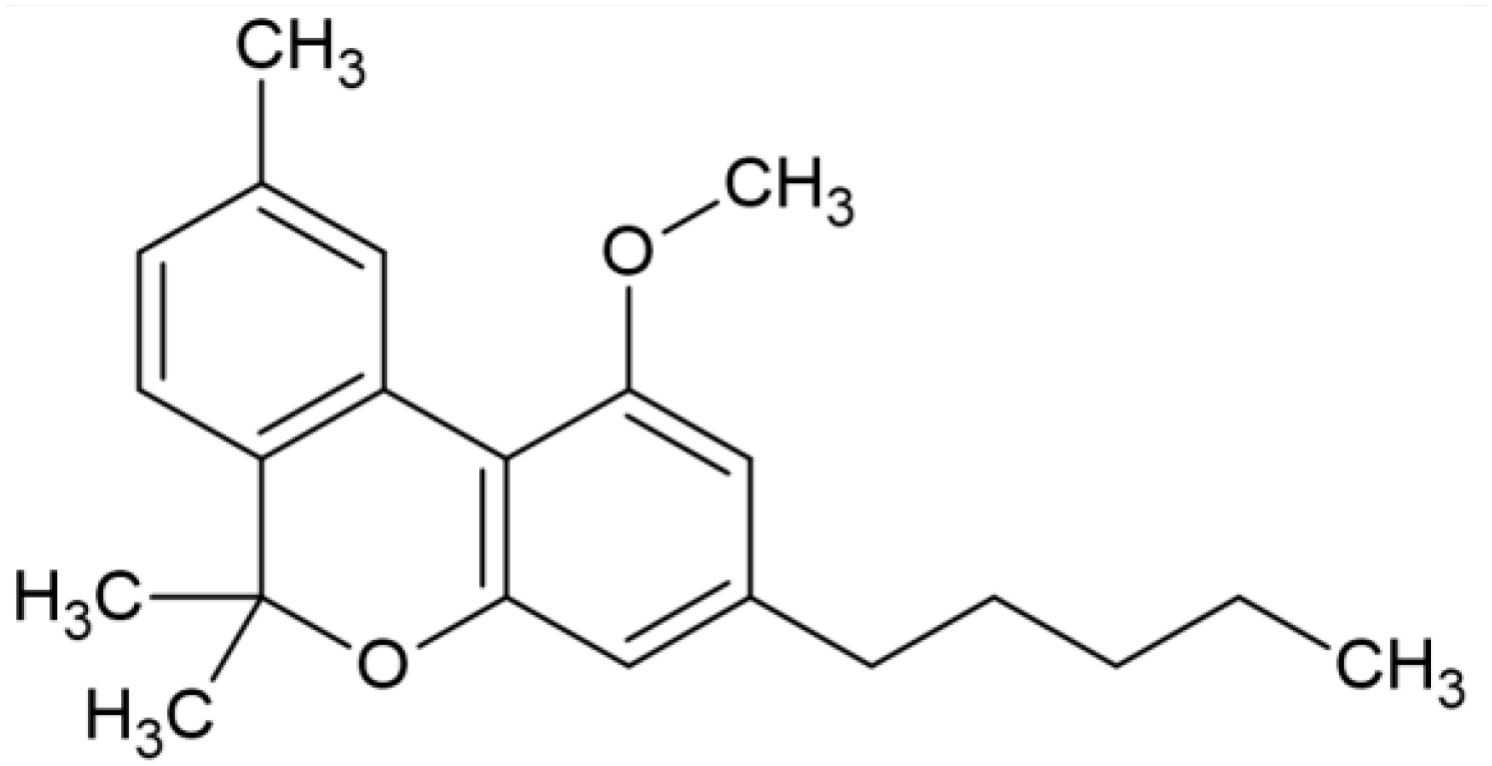
Cannabinol	1.8	28.1	43.9	1.6	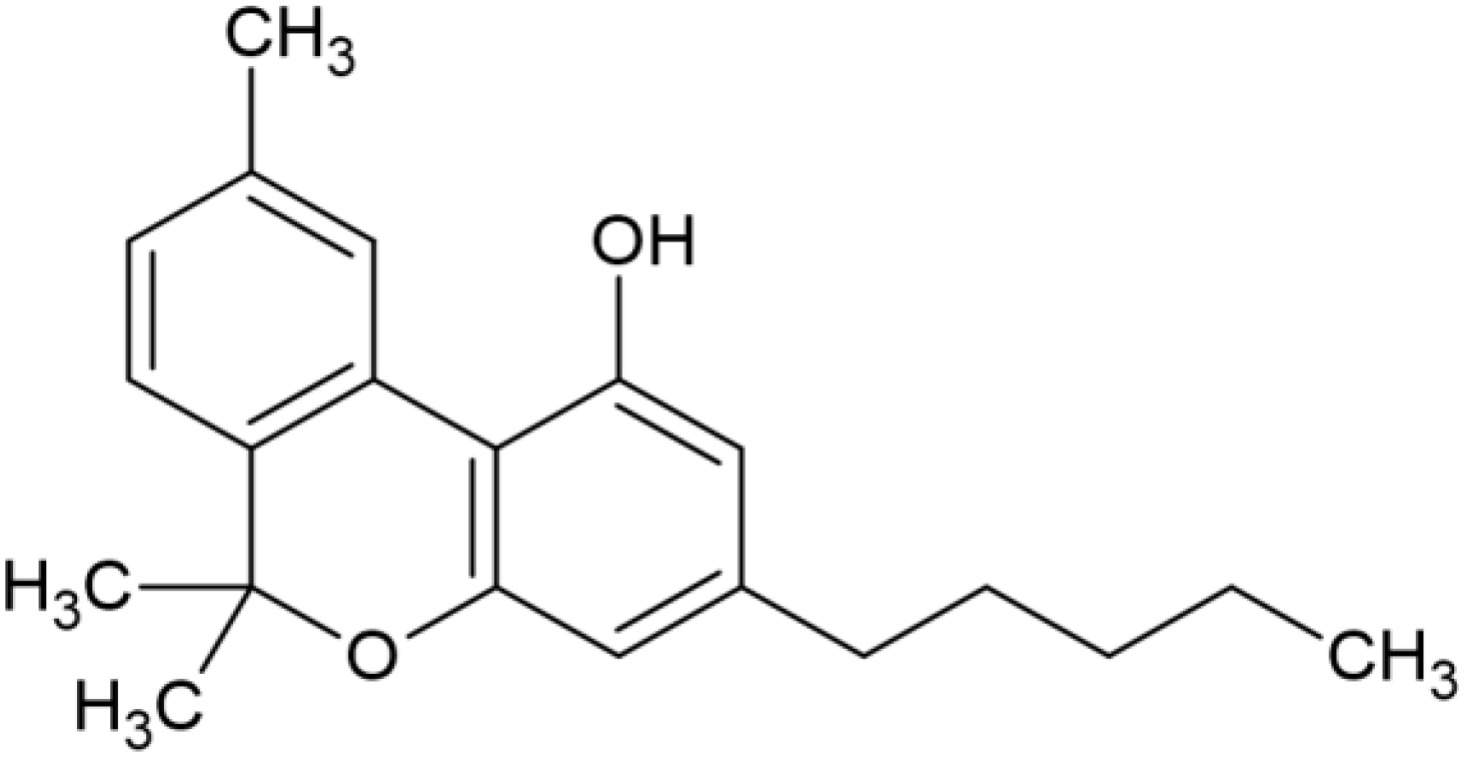
Cannabichromene	1.6	26.6	42.1	1.6	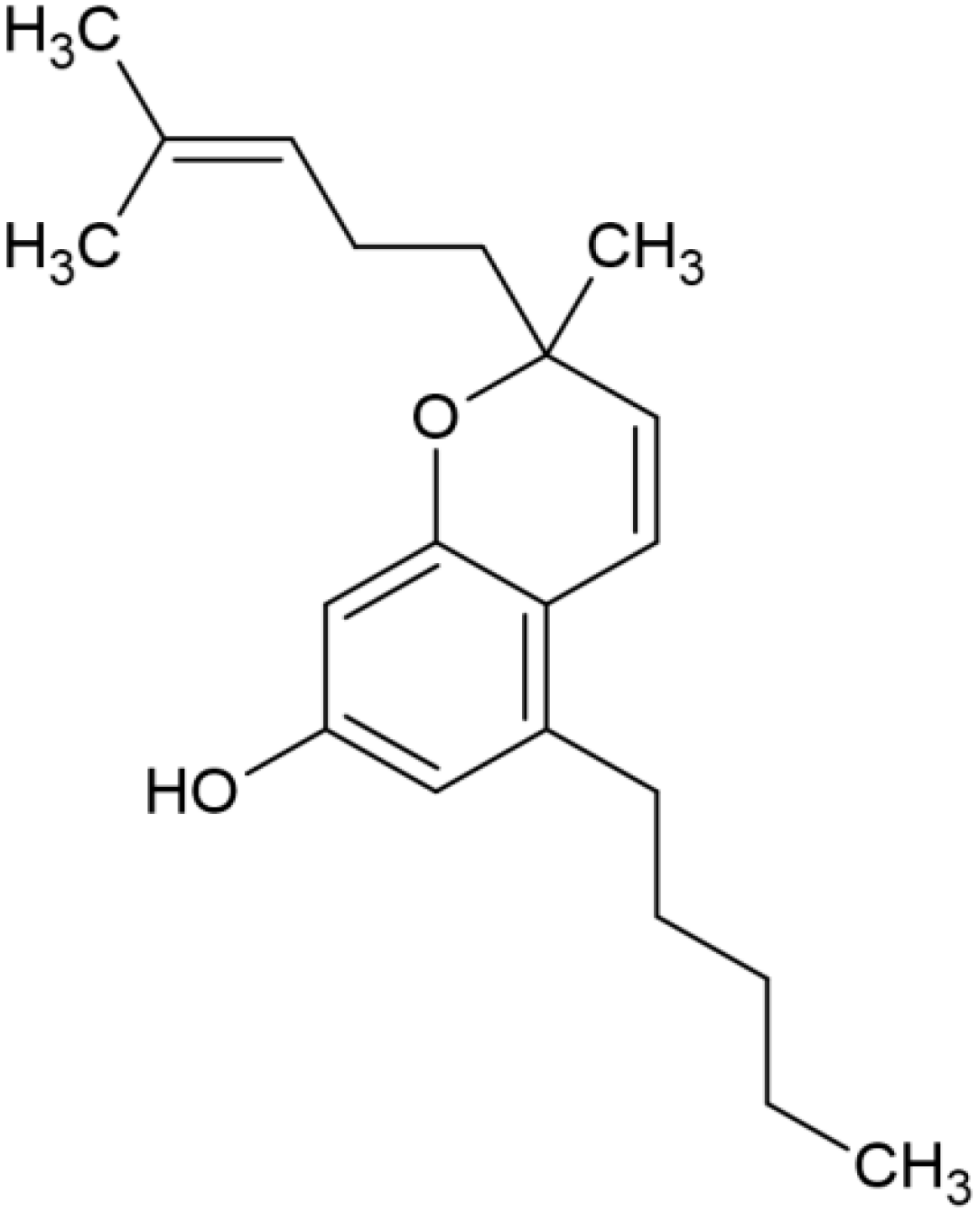
Cannabicitran	1	ND	NT	ND	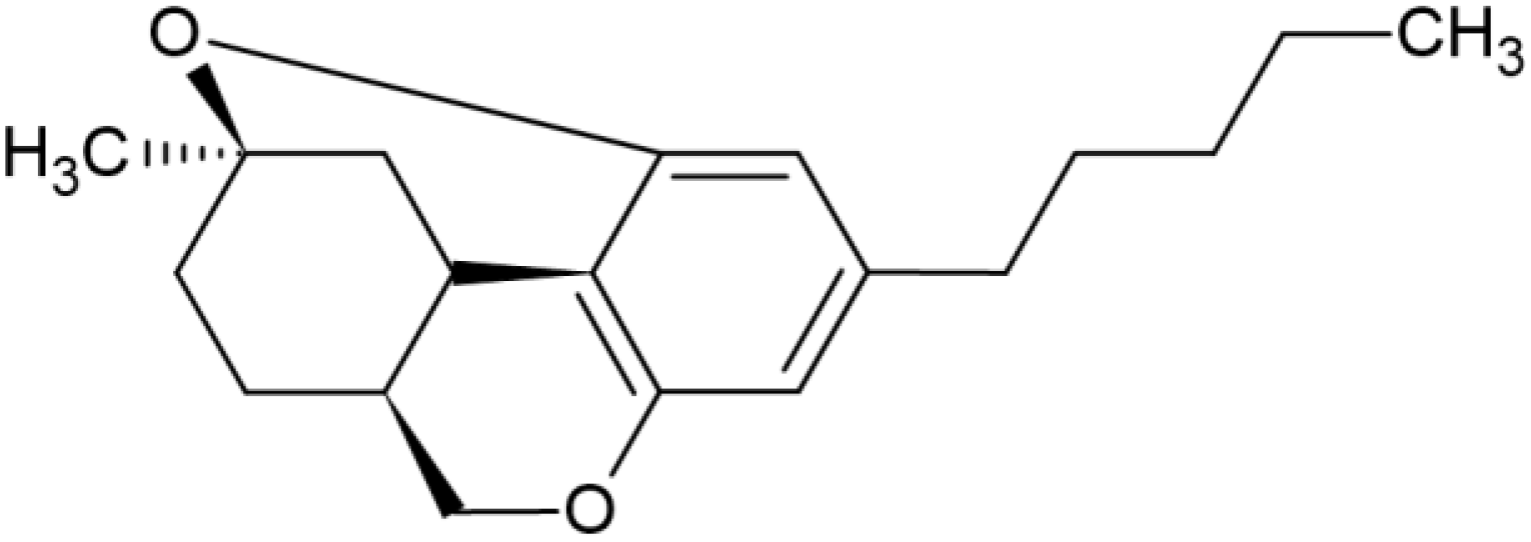

Fold induction, MFI of cannabinoid-treated cells at maximum induction/MFI of vehicle-treated cells.

EC_50_, effective concentration, 50% of maximum.

LC_50_, lethal concentration, 50% of maximum.

Selectivity Index, LC_50_/EC_50_.

NT, not toxic at concentrations tested.ND, Note determined.

### Specific synthetic cannabinoids can induce MHC-I expression in metastatic cancers

Over time, numerous compounds have been synthesized to interact with and modulate the endogenous cannabinoid receptors, either as agonists, inverse agonists, or antagonists (48, 49). A library of 371 synthetic cannabinoids was screened to determine whether any of the compounds share the capacity of phytocannabinoids to induce MHC-I expression on COLO 205 cells. A pilot experiment on a subset of the library demonstrated that a number of the compounds were active in the range of 20-40 µM, so the entire library was initially screened at a single concentration (35 µM) for each compound. This screen showed that many of the synthetic cannabinoids can induce MHC-I expression in COLO 205 cells, with 53 of them achieving a three-fold or higher level of induction (**Table 3**).

The synthetic compounds in the library can generally be grouped into seven families based upon structural similarities (**Tables 1**, **3**). All compounds within each family with at least a 3-fold level of MHC-I induction in the initial screen, as well as several compounds that did not reach the 3-fold threshold at 35 μM were tested in dose titrations spanning 6.8 to 100 μM in the COLO 205 assay. The EC_50_ values from these curves revealed that several of the synthetic cannabinoids are as potent as CBD. Structures of representative members of each structural family are illustrated in **Table 1**. While some structural similarities are apparent between the distinct synthetic families, and between the synthetic families and the phytocannabinoids, they are all dissimilar to the endocannabinoids. Also notable, most of the cannabinoids, both synthetic and plant-derived, show a bell-shaped MHC-I induction curve (**Figure 4**; **Table 1**), in which induction diminishes as cannabinoid concentrations increase past the maximum induction point. In most cases, this diminishment correlates with increasing toxicity of the compound to the cells. All compounds tested are listed by family with fold induction data in **Supplemental Table 1**.

**Table 3:** MHC-1 induction in COLO-205 cells by synthetic cannabinoids.

Family	Molecular Weight	Compounds in Family	Compounds with ≥3X MHC-I Induction	Range of Induction (Fold)	EC_50_ (µM)
1	300 – 500	131	20	0.6 – 5.6	13
2	300 – 410	85	14	0.5 – 6.8	13
3	330 – 440	67	13	1.1 – 7.0	27
4	500 – 550	2	2	4.0 – 5.8	23
5	300 – 390	23	1	0.9 – 3.2	10
6	290 – 420	15	1	1.1 – 4.2	23
7	295 – 415	26	2	0.17 – 3.17	ND

Fold induction, MFI of cannabinoid-treated cells at maximum induction/MFI of vehicle-treated cells.

EC_50_, effective concentration, 50% of maximum, of most potent cannabinoid in each family.

ND, not determined.

### Metastatic carcinomas treated with cannabigerol or IFN-γ reconstitute antigen processing recognized by MHC-I restricted T-lymphocytes

We next tested whether treating metastatic tumours with cannabinoids and pulsing them with the peptide that is recognized by a clonotypic T cell receptor expressed in MHC-I restricted CTL can facilitate recognition of the metastatic tumour (50–53). We have used this method together with the CFSE dilution assessment of T lymphocyte recognition and proliferation, that is an alternative to Chromium release assays (54). Furthermore, this assay is used here as a proxy for recognition of antigen presentation of “tumour-specific” antigens in the context of MHC-I molecules by tumour-specific CTL.

**Figure 5:**
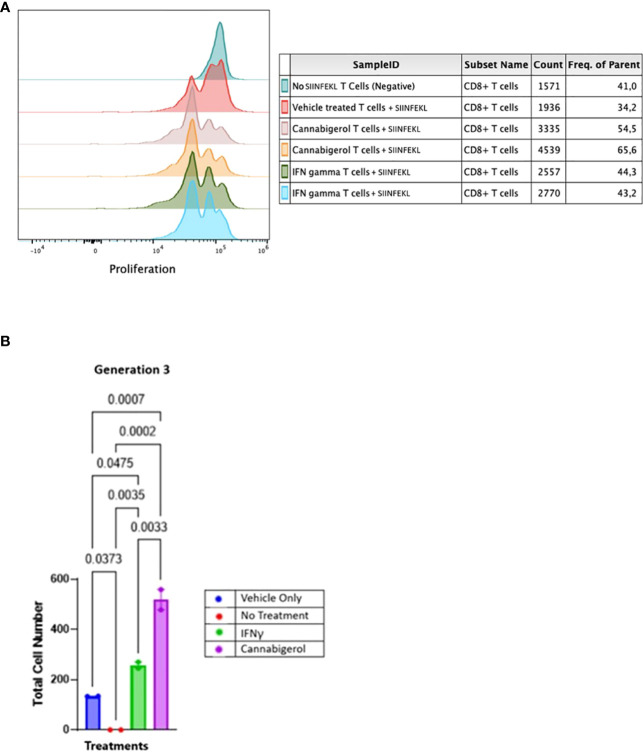
Cannabinoids treated metastatic carcinomas function as antigen presenting cells. **(A)** We used CD8^+^ T lymphocytes from SIINFEKL-primed OT1 mice that recognize and respond to SIINFEKL peptide presented on MHC class I of metastatic Murine A9 lung carcinomas. A9 cells were treated with 0.055 μmol of Cannabigerol, or 5.832x10^-6^ nmol mL of IFN-γ used as a positive control. The negative control is CD8^+^ T cells alone or untreated A9 cells pulsed with the SIINFEKL peptide from ovalbumin. T cells were labeled with CFSE proliferation dye, which is reduced within the OT1 progeny cells as the generation number increases as an indication of proliferation. **(B)** Statistical assessment based using the one-way ANOVA with Tukey’s multiple comparison test of CFSE assay demonstrates both cannabinoids and IFN-γ resurrect antigen presentation in metastatic carcinomas. A P value smaller than 0.05 was considered significant. Analysis of the CFSE proliferation carried out at cellular proliferation generation 3 demonstrates that both cannabinoid and IFN-γ treated CFSE contain OT-1 CD8^+^ T lymphocytes proliferated significantly more than the OT-1 CD8^+^ T lymphocytes alone or untreated A9 cells pulsed with SIINFEKL.

In order to assess whether MHC-I induction by cannabinoids has the potential to enhance CD8^+^ CTL recognition of a cancer cell, we performed a CFSE T lymphocyte proliferation assay. As we have shown previously (31), an increase in H-2K^b^ on A9 cells corresponds with an increase in the tumor cells’ presentation of antigen to CD8^+^ T lymphocytes. In the present experiment, this elevated antigen presentation stimulates a concomitant increase in OT1 mouse (ovalbumin-peptide SIINFEKL-specific, H-2K^b^ restricted) CD8^+^ T lymphocytes proliferation is indicated by the successive peaks of CFSE dilution. *Ex vivo*, OT1 mouse CD8^+^ T lymphocytes were found to increase in proliferation following their co-culture with H-2K^b^ A9 metastatic tumours treated with SIINKFEKL and cannabigerol or IFN-γ for 48 hours prior to co-culture, suggesting that the OT-1 CD8^+^ T lymphocytes are activated, which may be expected to result in cytolytic activity against these cells (**Figure 5**).

The data was analyzed further as described in earlier studies (55). The cell number resulting from OT-1 CD8^+^ T lymphocytes proliferation was counted and plotted. The data from all the groups at different generation times was plotted in GraphPad prism and Gaussian distribution was applied. IFN-γ and cannabigerol treated antigen presenting cell groups showed Gaussian distribution in triggering CSFE loaded OT-1 CD8^+^ T lymphocytes to proliferate. To compare the OT-1 CD8^+^ T lymphocytes proliferation from treatment groups, the total cell number from the different treatment groups at each cell generation was plotted and compared using one way ANOVA with Tukey’s multiple comparison test. Any P value smaller than 0.05 was considered significant. At generation 1, neither the cannabigerol nor IFN-γ-treated antigen presenting cells triggered OT-1 CD8^+^ T lymphocytes proliferation greater than the vehicle treatment. However, at generation 3, both IFN-γ and cannabigerol treated antigen presenting cells triggered T cells proliferation significantly more than the vehicle treated group (**Figure 5B**; **Appendix Figure 1**).

### Cytokine profile in cannabigerol treated metastatic murine carcinomas

**Figure 6:**
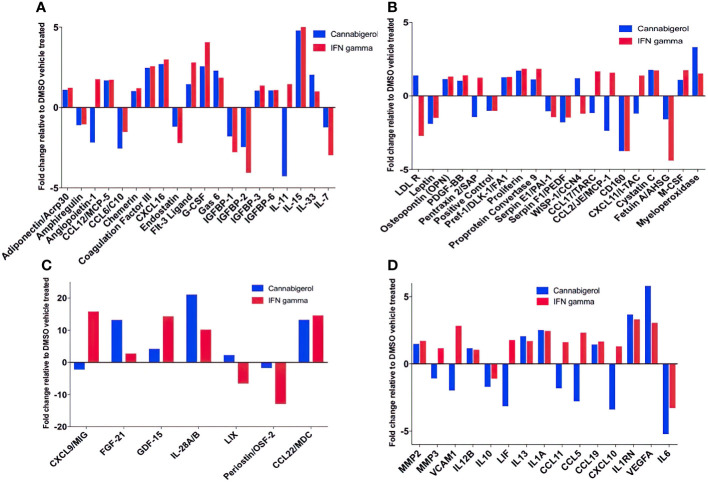
Fold change of cytokine production in Metastatic Carcinomas treated with cannabigerol or in supernatant upon treatment of 0.00875 mg/mL cannabigerol (0.05529 μmol) and 0.1ug/mL (100ng/mL) (5.832x10^-6^ nmol) IFN-γ relative to DMSO (vehicle)-treated metastatic A9 cells. **(A–C)** Fold change of cytokines present on microarray **(D)** Cytokines implicated in the IL4, 10, and 13 pathways. Data was determined using Proteome Profiler Mouse XL cytokine array kit and Image J protein analyzer add-on. Pathway analysis was determined using Reactome Database release 65, Pathway Brower Version 3.5.9.

Next, changes in the expression of chemokines, cytokines and related molecules in response to cannabigerol and IFN-γ treatment were explored. Both treatments were shown to have similar effects on a range of immune markers, including the upregulation of IL-28A/B, CCL22, FGF-21 (**Figure 6C**), and most interestingly IL-33 (**Figure 6A**) and the downregulation of IL-6 (**Figure 6D**), and IL-11 (**Figure 6A**). Finally, there was an increase in VEGFA (**Figure 6D**). Cannabigerol was also found to cause a change in cytokines involved in inflammation, migration, growth and differentiation, angiogenesis, immune regulation, leukocyte development and metabolism (**Figure 6**). However, certain markers were differentially regulated by the treatments, offering insights into the potential anti-cancer mechanisms specific to cannabigerol and IFN-γ. Cannabigerol-specific effects included the inhibition of angiopoietin-1, MMP3 and VCAM-1, indicating that its anti-cancer effects may be mediated by the modulation of vascular-immune interactions. Angiopoietin-1 is a secreted glycoprotein that binds to endothelial cell-specific tyrosine-protein kinase receptors to promote in vascular development and angiogenesis. Matrix metalloproteinase 3 (MMP3) is a protein involved in the degradation of components of the extracellular matrix (fibronectin, laminin, collagens III, IV, IX, and X, and cartilage proteoglycans), with a known role in tumour initiation (56). Vascular cell adhesion molecule 1 (VCAM-1) is a cell surface sialoglycoprotein expressed by cytokine-activated endothelium, important for adhesion of leukocytes to endothelial cells and subsequent signal transduction. Angiogenesis and endothelial cell adhesion are generally thought to promote tumour formation and migration (57). On the other hand, treatment with IFN-γ, but not cannabigerol, was associated with a reduction in low density lipoprotein receptor (LDLR), which may be an additional anti-cancer mechanism specific to IFN-γ (58). Finally, these data suggest that cannabigerol and IFN-γ exert their anti-cancer properties *via* the inhibition of STAT3 (upregulated by IL-11 and LIF) and c-Jun/AP-1 (downregulated by Pentraxin2/SAP), respectively.

### Functional annotation of H3K27ac marks induced by cannabinoids in an antigen processing deficient metastatic carcinoma

**Figure 7:**
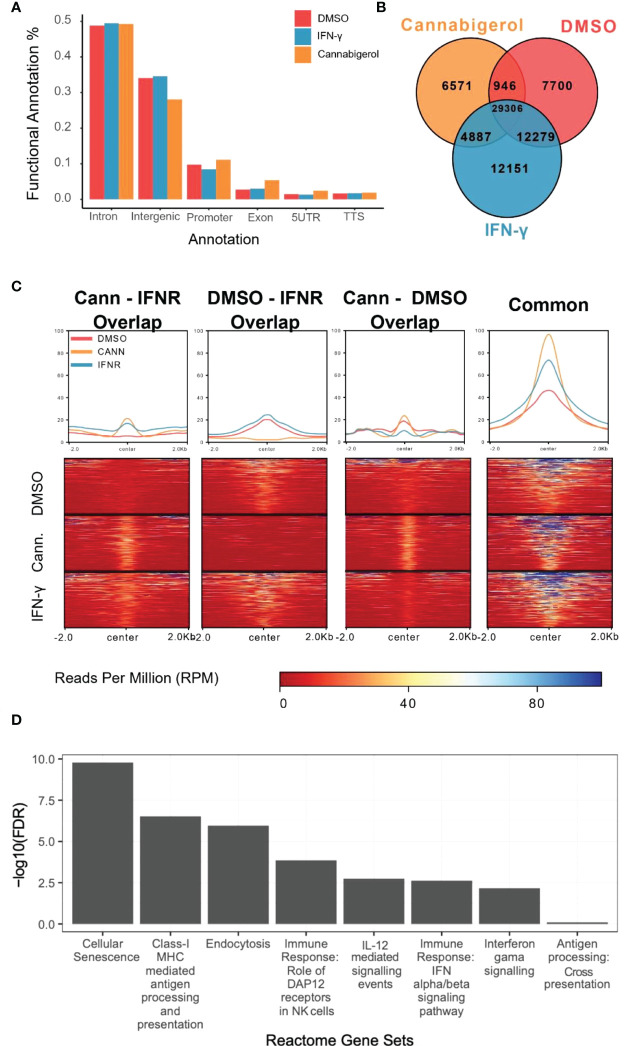
Functional annotation of H3K27ac marks induced by cannabinoids in an antigen processing deficient metastatic carcinoma. **(A)** Gene regions modified by H3K27ac. Regions were plotted. **(B)** H3K27ac peaks locations were compared. **(C)** H3K27 acetylation levels of common regions were higher than all other combinations of interactions. At common regions, cannabigerol induction demonstrates increased global acetylation similar to IFN-γ. **(D)** Pathway analysis of closest genes with respect to overlap of Gained/Cannabigerol gene sets < 0.01 FDR were filtered out. Functional Annotation of H3k27ac regions showed that cannabigerol and IFN-γ acetylation profile enriched on intergenic/intronic parts of the genome. DMSO and IFN-γ samples then annotated as DMSO only (Lost, n= 8588), DMSO-IFN-γ common (Common, n=39311) and IFN-γ only (Gained, n= 15886). DMSO and cannabigerol samples then annotated as DMSO only (n= 15972), DMSO-cannabigerol common (n=31927) and cannabigerol only (Gained, n= 16746).

H3K27Ac epigenetic modifications are generally associated with transcriptional activation of gene and H3K27Ac ChIPseq is an established method to identify genes and pathways that are induced following treatment with a drug. To understand how HDAC activators can potentially alter immune evasion *in situ* we conducted H3K27Ac ChIPseq on DMSO, IFN-γ and cannabigerol treated cells. Functional Annotation of H3K27ac regions from all samples demonstrated that the most significant alterations in histone modifications were observed at intronic and intergenic sites suggesting cannabigerol and IFN-γ alter H3K27Ac at enhancer sites (**Figure 7A**). We also found 40% of acetylation marks were located in H3K27ac regions which are observed commonly in all samples (**Figure 7B**). Interestingly, in this common region set, we observed that the effects of cannabigerol were similar to IFN-γ suggesting that both cannabigerol and IFN-γ increase overall acetylation levels to initiate immune response (**Figure 7C**). A pathway analysis of closest genes with respect to overlap of Gained/Cannabigerol gene sets < 0.01 FDR were filtered out (**Figure 7D**). These included cell senescence, Class-I MHC mediated antigen processing and presentation, immune response genes related to DAP12 receptors in NK cells. IL-12 mediated signaling events, interferon alpha (IFN-α) and interferon beta (IFN-β) and gamma (IFN-γ) signaling pathways and antigen processing cross-presentation pathway genes were all enriched. This reinforces the observation that cannabigerol can reverse the immune-escape phenotype in metastatic tumours.

## Discussion

Cannabinoids have demonstrated biological and pharmacological effects, including pain reduction, inhibition of nausea, appetite induction, anxiety and depression reduction, among others (59). Some of these activities are of benefit in cancer therapy, especially for reducing nausea, pain, and depression, but also for increasing appetite (60). While there are some reports of a direct cytotoxic effect of cannabinoids on tumor cells (61), there are no publications that demonstrate MHC-I induction by cannabinoids. Given the public interest in this area, the identification of cannabinoids that possess “immune escape” reversing activities may have significant impact on cancer immunotherapy and wellness seeking.

Understanding the mechanisms that promote cancer metastasis is profoundly important, as metastatic cancers account for 90% of all cancer deaths (1). The cellular immune system plays an essential role in reducing cancer progression through immune surveillance. In the absence of functional antigen processing machinery, adaptive immune responses fail to limit the emergence of tumours. During endogenous antigen processing, resident proteins are broken down to peptides and loaded onto MHC-I molecules. These subsequently cycle to the plasma membrane, peptide in tow, to present their cargo to T cell receptors (TCR) expressed by CTL. The TCR recognize the precise combination of specific the peptide bound to MHC-I molecules with exquisite accuracy. To generate the peptides, exogenous proteins are degraded by proteasomes in the cytosol before being transported into the ER by TAP-1 and -2. In the ER, as a result of the concerted action of a number of molecular chaperone proteins, the peptides are loaded onto the MHC-I molecules before being transported to the cell surface (1, 22–27). Overall, this mechanism antigenically defines self and non-self, thereby allowing CTL to distinguish between normal cells and cancerous or virus-infected cells. Following this interaction that provide a cue for activation of the effector functions of CTL, a specific immune response can be initiated, which generally leads to the destruction of the cancerous or virus infected cells (23, 62, 63) but may also act as a powerful selective force for the diabolical emergence of virus or tumour antigen escape mutants (1, 22–27).

Many cancerous cells display down-regulated MHC-I cell surface expression but do not possess structural mutations in either MHC-I genes or β2-microglobulin (41, 64–67). Reduced MHC-I expression can result at least in part from the downregulation or mutation of other genes such as transporters (for example, TAP-1, TAP-2), proteasome components (LMP), and other accessory proteins involved in the antigen presentation and processing pathway. However, immune escape is not exclusively regulated by defects or mutations in APM genes but can also be epigenetically regulated and can be restored by treatment with histone deacetylase inhibitors (HDACi), such as TSA (29, 31). With this in mind, we conducted a screen and found that (i) cannabinoids can reverse the immune escape phenotype of both human and murine metastatic tumours and (ii), metastatic tumours induced by cannabinoids can upregulate MHC-I expression and act as MHC-I antigen presenting cells to promote CD8^+^ T lymphocytes proliferation *in vitro*.

The molecular mechanisms linking cannabinoid administration to MHC-I induction remain to be fully defined. Cannabinoids are known to modulate G protein-coupled receptors (GPCR), transient receptor potential channel, and voltage-dependent membrane channel activity (33, 68, 69). We found that engagement of the cannabinoid receptors, CB1R or CB2R, *per se* does not activate the MHC-I pathway, as neither of the endogenous cannabinoids, 2-AG and AEA, induced MHC-I expression by COLO 205, which expresses both cannabinoid receptors (70). This suggests that other receptors also associated with cannabinoid signaling may be involved, such as the GPCRs GPR3, GPR6, GPR12, GPR18 and GPR55, serotonin receptors 5-HT1A and 5-HT2A, μ- and δ-opioid receptors, and the adenosine A3 receptor (68). Phytocannabinoids can also activate transient receptor potential channels of the vanilloid subtype and voltage-gated sodium channels, which are expressed in various cancers. Cannabinoids also inhibit voltage-gated calcium channels, specifically the Cav1 and Cav3 families (69). However, the fact that low micromolar concentrations of cannabinoids are required to induce MHC-I suggests that the molecules may act through a non-receptor-mediated mechanism. The distinct induction kinetics displayed by IFN-γ and CBD suggest that different pathways are invoked, although it is possible that IFN-γ acts downstream of CBD en route to MHC-I induction. The long lag period of 48 hr before robust MHC-I upregulation suggests that the induction by CBD depends upon activation of new gene expression. Consistent with this possibility, low micromolar levels of CBD have been found to regulate expression of cellular stress response genes in microglial and lung epithelial cells (71, 72), genes involved in neurotransmitter signaling in neural cells (73), and genes associated with cell proliferation and division and DNA repair in squamous cell carcinoma cells lines in head and neck cancers (74). Interestingly, in a study examining gene expression in the A549 lung epithelial cell line infected with SARS-CoV-2, CBD was found to reverse the changes in gene expression induced by the virus and to upregulate genes that promote innate immunity such as receptors for IFN-γ and IFN-β and the signaling proteins STAT1 and STAT2 that transduce the interferon signal (71). In a study performed by van Breeman et al. (75), cannabigerolic acid (CBG-A) and cannabidiolic acid (CBD-A) prevented infection of human epithelial cells by a pseudovirus expressing the SARS-CoV-2 spike protein and prevented entry of live SARS-CoV-2 into cells.

In this study, other cytokines of importance to cancer that were upregulated upon the use of cannabigerol included IL-28A, CCL22, FGF-21 (**Figure 6C**), and IL-33 (**Figure 6A**). IL-33 is termed an “alarmin” and its expression is associated with the upregulation of MHC- I and APM (30, 31) and is decreased during metastasis (30, 31). The loss of IL-33 expression is also a predictor of poor outcome in kidney and prostate carcimonas (30, 31). In the same context, it was observed that normal epithelial cells and MHC-I+ primary tumours express IL-33 (31) and endogenous IL-33 acts in an autocrine loop to induce MHC-I expression thereby insuring immune surveillance of normal epithelial cells and limiting the emergence of tumours by surveying primary tumours as well. The transcriptional link between these two genes has also been previously demonstrated by genetic complementation experiments where a recombinant IL-33 gene was reintroduced into metastatic cells resulting in a rescue of MHC-I expression and tumour recognition by CTL and reduction of tumour growth *in vivo* (30). Furthermore, IL-33 is also known to be the hallmark cytokine for activating Group 2 innate lymphoid cells (ILC2s). We subsequently demonstrated ILC2 can mediate and enhance T_H_1 CTL responses and are directly involved in tumour immunosurveillance and elimination in mice (30). Once ILC2s are functionally activated, they alter the tumour microenvironment triggering both innate and adaptive immune responses. We used genetic studies to conclusively demonstrate that ILC2s dramatically reduce the number of circulating tumour cells, resulting in the reduction of the metastasis of tumours (30). These studies established a new, hiterto undescribed, form of immune escape mechanism involving the loss of IL-33 and muting ILC2 function in T_H_1 responses. The data in the present study describes the ability of cannabigerol to increase IL-33 expression and uncover a potential method by revive IL-33 expression and ILC2 function leading to enhanced CTL responses against tumours. Additionally, CCL22 is usually secreted in response to IFN-γ and TNF alpha or IL-4 (35) and is associated with the induction of chemotaxis of T lymphocytes by the binding to CCR4. The production of CCL22 by cannabigerol-treated metastatic cells could provide a method by which the T lymphocytes could be recruited into the tumour site.

Interestingly, we observed downregulation of IL-6 in cannabigerol-treated metastatic A9 cells (**Figure 6D**). IL-6 is associated with differentiation of naïve CD4^+^ lymphocytes against a specific antigen, and of differentiation of CD8^+^ naïve cells into CTLs (36). Also of note, the cannabigerol-treated A9 cells also appeared to produce IL1RN, an IL-1 antagonist (37) and negative regulator of inflammation, providing an additional avenue for explaining the anti-inflammatory role of cannabinoids. Notably, There was also an increase in VEGFA expression in cannabigerol-treated A9 cells. VEGFA is a crucial gene for the formation of blood vessels and angiogenesis (VEGFA NIH) (38), during which the new blood vessels supply nutrients to a tumour and increase tumorigenesis (3). In the future, it might be interesting examine if angiogenesis is altered in cannabigerol treated mice.

To further understand the mechanism by which cannabinoids may act to reverse immune escape of metastatic carcinomas, we conducted H3K27Ac ChIPseq study to examine H3K27Ac, an epigenetic mark associated with gene activation. Following treatment with a cannabigerol or IFN-γ. Functional annotation of regions marked by H3k27ac showed that both cannabigerol and IFN-γ acetylation profile enriched on intergenic/intronic parts of the genome. A common gene set was shared by cannabigerol and IFN-γ induction suggesting cannabinoids may share some of the attributes of IFN-γ. Finally, gene enrichments analysis highlighted genes involved in cell senescence, MHC-I mediated antigen processing and presentation, and immune response genes related to DAP12 receptors in NK cells. IL-12 mediated signaling events, IFN-α, IFN-β, and IFN-γ signaling pathways and antigen processing cross-presentation pathway genes were all enriched. This reinforces the observation that cannabinoids can reverse the immune-escape phenotype in metastatic tumours and supports the somewhat surprising conclusion that, in many ways, cannabigerol acts like IFN-γ, a master-regulator of T_H_1 responses.

While our data advance the potential of cannabinoids to reverse the immune editing and escape that are characteristic of metastatic cancer, two important factors warrant consideration. First, relatively high concentrations of cannabinoids are needed to induce MHC-I expression – *e.g.*, an EC_50_ of 11 μM for CBD on COLO 205 cells – suggesting that patients will require high doses for the effect to manifest. Such levels of CBD should be safely achievable, as up to 45 μM CBD have been reported in the plasma of mice without severe adverse consequences (76). High dosing is also well tolerated in humans, with subjects receiving up to 1500 mg/d of CBD reporting only mild adverse effects (77–80).

The second factor to consider is that cannabinoids have been reported to have anti-inflammatory and immunosuppressive properties. This raises the possibility that any pro-immunosurveillance benefit of cannabinoid treatment might be countered by a detrimental immunosuppressive effect. Some Cannabinoids have been reported to reduce antibody and T Lymphocyte responses and increased susceptibility to infection (81–85). In several *in vitro* and *in vivo* models of infection and autoimmunity, THC, CBD, cannabinol, and synthetic cannabinoids have all been found to alter immune function (86–88). These data should be considered with caution, however, because the source and physiological context from which experimental cells are derived may significantly impact how they respond to treatment. For example, peripheral blood mononuclear cells (PBMCs) from multiple sclerosis patients were found to be more sensitive to the anti-proliferative effect of CBD or THC than PBMCs from normal or cancer patients (89). Furthermore, since nearly all the studies demonstrating anti-inflammatory and immunosuppressive properties of cannabinoids were conducted in cell culture or in rodent models, there is no clear demonstration of immunomodulatory effects of cannabinoids in humans (85). In fact, as noted above, extensive clinical testing of high dose CBD (Epidiolex) in patients with Lennox-Gastaut or Dravet syndromes, two rare, severe forms of epilepsy, found minimal evidence of compromised immunity (77, 78). Similarly, cancer patients treated with pharmacologically active doses of the synthetic THC analogs, dronabinol and nabilone, for chemotherapy-induced nausea or anorexia showed no signs of reduced immunity (90–94). Likewise, HIV patients treated with dronabinol for HIV wasting syndrome exhibited no increase in opportunistic infections (95). Even advanced HIV patients at elevated risk for opportunistic infections and treated with dronabinol to ameliorate anorexia showed no greater incidence of infection than non-treated patients (96). A possible immunologically beneficial effect of THC treatment in HIV subjects was noted in a non-human primate model: chronic administration of THC to male rhesus macaques infected with simian immunodeficiency virus (SIV; model for HIV infection) resulted in decreased viral load and increased lifespan compared to control animals (97). With respect to cancer, several studies have shown that cannabinoids preferentially inhibit or kill human cancer cells *in vitro* (98). In a xenograft mouse model used to study head and neck squamous cell carcinoma, CBD alone slowed tumor growth and synergized with cisplatin for a dramatic delay in tumor growth (74). Whether this translates to a benefit for patients remains to be seen. Nevertheless, the preponderance of evidence indicates that administration of cannabinoids to cancer patients is unlikely to result in generalized immune suppression, supporting the possibility that the immunosurveillance-promoting activity of these molecules will prevail in these patients.

Recent advances in immunotherapy have significantly improved outcomes for some patients with cancer. Combination therapy of antibodies against the antagonistic co-inhibitory receptor Programmed death-1(PD-1) (99) and agonistic OX40 (100), that are currently under investigation (101). Likewise, our data suggest that cannabinoids, similar to complementation with TAP genes (1) may potentiate immune-checkpoint blockade inhibitor activity by reversing immune escape and restoring tumour visibility to the adaptive immune system. The investigation of pure cannabinoid molecules rather than plant extracts or formulations in combination with immune- checkpoint blockade inhibitors may facilitate the use of cannabinoids in clinical practice.

## Data availability statement

The datasets presented in this study can be found in online repositories. The names of the repository/repositories and accession number can be found below: The data discussed in this publication have been deposited in NCBI's Gene Expression Omnibus (102) and are accessible through GEO Series accession number GSE179897.

## Author contributions

Conceived Project: WAJ. Designed research: SD, LN, SE, CP, CW, RA, WAJ. Performed research: SD, LN, SE, NG, IS, LM, CD, PG, TM, NL, BE, RA, LT, EG. Analyzed data: SD, LN, SE, CD, NG, IS, LM, CP, SK, CW, PG, TM, NL, BE, RA, LT, GC, EAH, WAJ. Wrote paper: SD, SE, CW, WAJ. Edited paper: SD, LN, SE, CP, TM, NL, RA, GC, EAH, WAJ. All authors contributed to the article and approved the submitted version.

## Supplementary material

Appendix Figure 1 Composite depiction of cell proliferation data from different cell generations in treatment and control groups.

Appendix Figure 2 Significant increase in cell proliferation in generation 1 and 2 from the Cannabigerol treated group.

Supplementary Table 1 Family: grouping of compounds according to structure.
