# Supplementary figures and images for: Specific cannabinoids revive adaptive immunity by reversing immune evasion mechanisms in metastatic tumours
Source: Front Immunol. 2023 Feb 22;13:982082. doi: 10.3389/fimmu.2022.982082 (PMC10010394; doi:10.3389/fimmu.2022.982082)

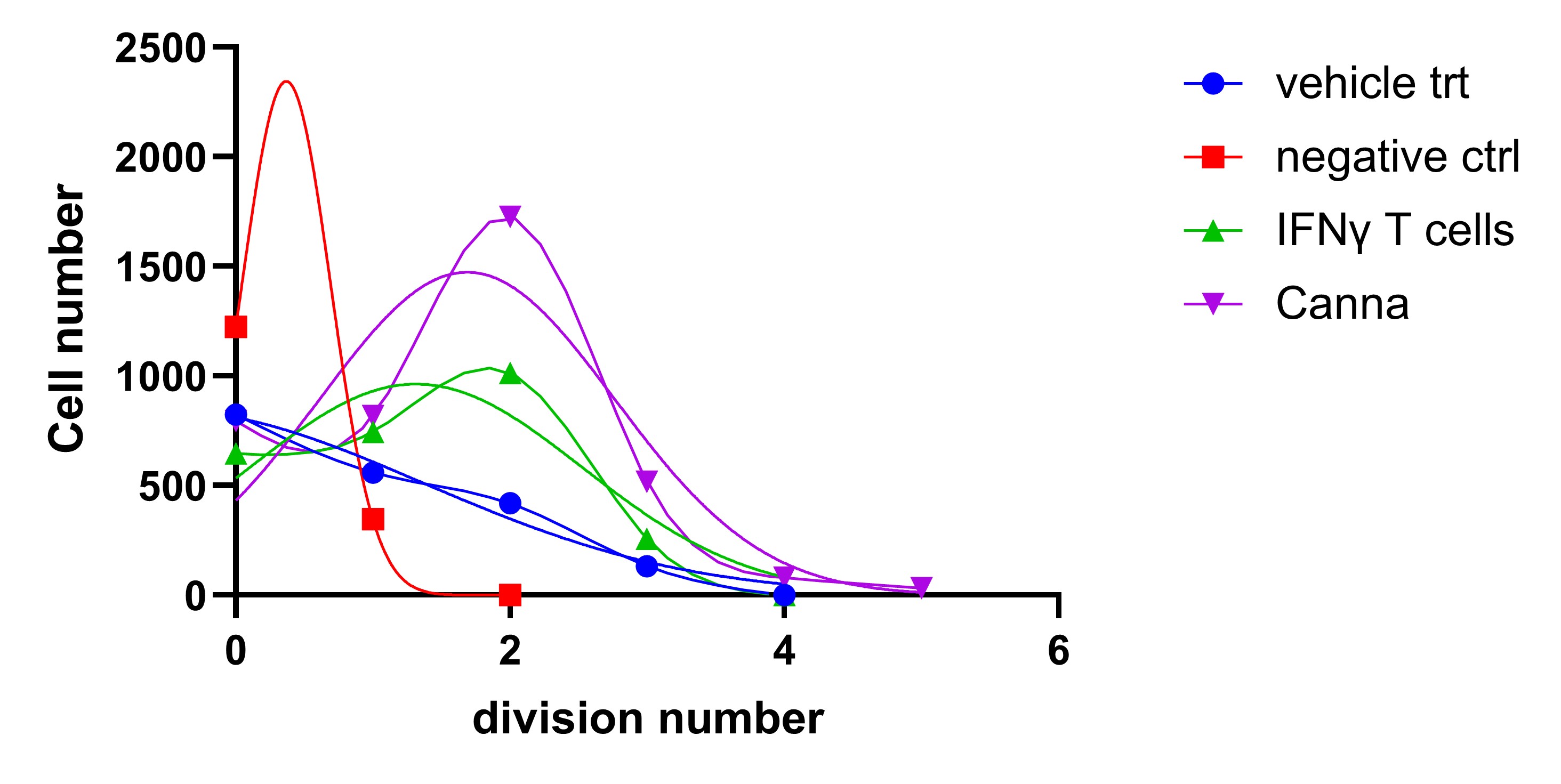

Supplement: Appendix Figure 1 — Composite depiction of cell proliferation data from different cell generations in treatment and control groups. [file Image_1.jpg]

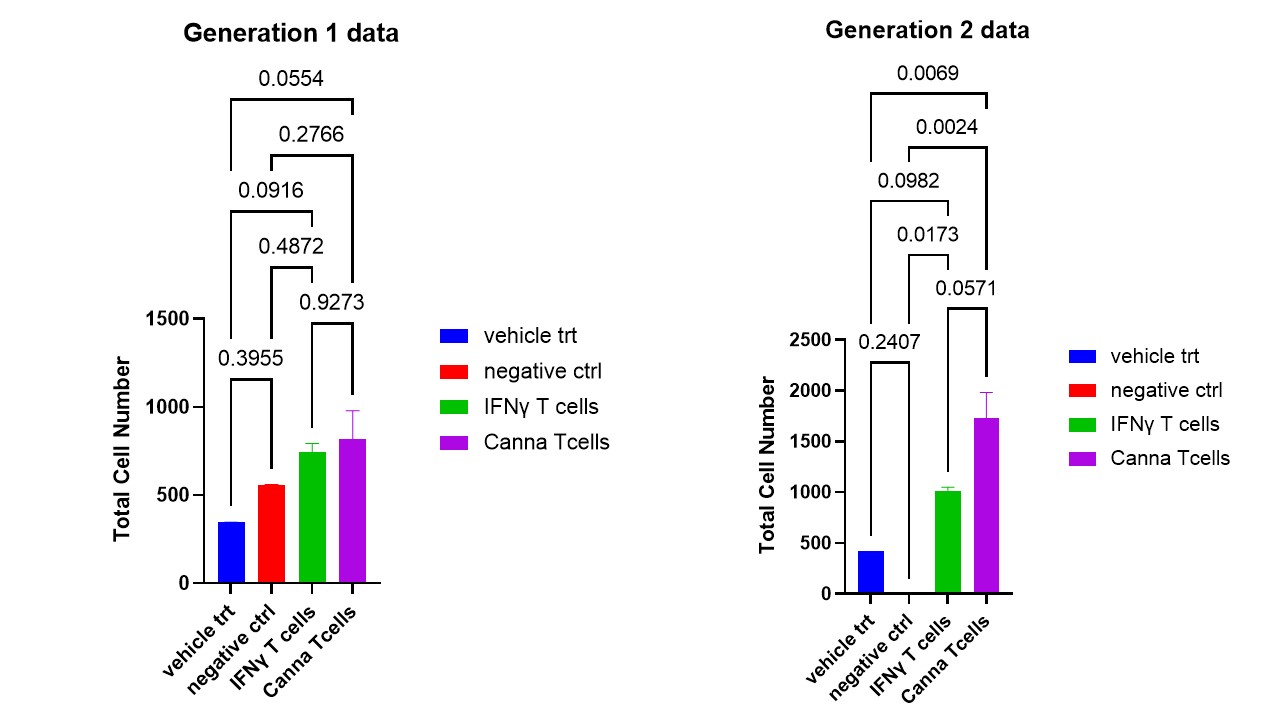

Supplement: Appendix Figure 2 — Significant increase in cell proliferation in generation 1 and 2 from the Cannabigerol treated group. [file Image_2.jpg]
