# Supplementary material for: Specific cannabinoids revive adaptive immunity by reversing immune evasion mechanisms in metastatic tumours
Source: Front Immunol. 2023 Feb 22;13:982082. doi: 10.3389/fimmu.2022.982082 (PMC10010394; doi:10.3389/fimmu.2022.982082)
Supplement: Supplementary Table 1 — Family: grouping of compounds according to structure. [file Table_1.docx]

**Supplemental Table 1:**

Family: grouping of compounds according to structure.

N/A: Compounds that do not have structures related to those of the seven defined families.

Plate and Well designations represent the location of each compound within the Cayman Chemical Co. synthetic cannabinoid library (#9002891)

MFI: mean fluorescence intensity; measure of MHC-I expression on target cells (COLO 205)

Viability: % of cells alive at time of assay

Fold Induction: MFI of cannabinoid-treated cells/MFI of non-treated control cells

| **Family** | **Plate** | **Well** | **Name** | Fold Induction |
| --- | --- | --- | --- | --- |
| 1 | 1 | A2 | JWH 007 | 2.4 |
| 1 | 1 | A4 | JWH 018 N-(5-chloropentyl) analog | 1.9 |
| 1 | 1 | A8 | JWH 081 | 1.6 |
| 1 | 1 | A9 | JWH 122 | 1.8 |
| 1 | 1 | B10 | AM2201 | 2.6 |
| 1 | 1 | B3 | JWH 182 | 2.0 |
| 1 | 1 | B4 | JWH 210 | 1.9 |
| 1 | 1 | B5 | RCS-4 | 2.3 |
| 1 | 1 | B6 | JWH 098 | 0.6 |
| 1 | 1 | B7 | JWH 018 N-(2-methylbutyl) isomer | 2.9 |
| 1 | 1 | B8 | JWH 018 N-(3-methylbutyl) isomer | 3.3 |
| 1 | 1 | B9 | JWH 018 6-methoxyindole analog | 1.9 |
| 1 | 1 | C5 | RCS-4-C4 homolog | 1.5 |
| 1 | 1 | D10 | RCS-4 3-methoxy isomer | 2.9 |
| 1 | 1 | D4 | JWH 073 2-methylnaphthyl analog | 3.8 |
| 1 | 1 | D5 | JWH 016 | 2.3 |
| 1 | 1 | D9 | RCS-4 2-methoxy isomer | 4.0 |
| 1 | 1 | E6 | JWH 018 | 2.7 |
| 1 | 1 | E7 | JWH 200 | 2.9 |
| 1 | 1 | E8 | JWH 073 | 3.3 |
| 1 | 1 | F6 | AM2233 | 3.2 |
| 1 | 1 | F7 | JWH 018 N-(5-bromopentyl) analog | 3.2 |
| 1 | 1 | F8 | JWH 018 N-(4,5-epoxypentyl) analog | 2.1 |
| 1 | 1 | G10 | JWH 018 2'-naphthyl-N-(1-ethylpropyl) isomer | 1.9 |
| 1 | 1 | G2 | AM1248 | 2.7 |
| 1 | 1 | G3 | AM2232 | 2.9 |
| 1 | 1 | G4 | AM679 | 3.3 |
| 1 | 1 | G7 | AM1220 azepane isomer | 5.6 |
| 1 | 1 | G8 | AM2233 azepane isomer | 3.6 |
| 1 | 1 | G9 | JWH 018 N-(1-ethylpropyl) isomer | 3.0 |
| 1 | 1 | H2 | JWH 122 N-(4-pentenyl) analog | 1.2 |
| 1 | 1 | H6 | JWH 213 | 1.7 |
| 1 | 2 | A10 | JWH 412 | 2.1 |
| 1 | 2 | A3 | MAM2201 N-(2-fluoropentyl) isomer | 1.0 |
| 1 | 2 | A4 | MAM2201 N-(3-fluoropentyl) isomer | 2.0 |
| 1 | 2 | A5 | MAM2201 N-(4-fluoropentyl) isomer | 2.2 |
| 1 | 2 | A7 | EAM2201 | 1.8 |
| 1 | 2 | A9 | THJ 018 | 2.1 |
| 1 | 2 | B2 | JWH 387 | 1.9 |
| 1 | 2 | B4 | MAM2201 N-(5-chloropentyl) analog | 1.9 |
| 1 | 2 | C10 | JWH 149 | 1.2 |
| 1 | 2 | C3 | JWH 019 | 2.2 |
| 1 | 2 | C5 | JWH 398 | 1.8 |
| 1 | 2 | C7 | JWH 071 | 1.5 |
| 1 | 2 | C9 | JWH 116 | 2.1 |
| 1 | 2 | D11 | JWH 146 | 1.8 |
| 1 | 2 | D2 | JWH 193 | 1.9 |
| 1 | 2 | D3 | JWH 198 | 3.8 |
| 1 | 2 | D4 | JWH 080 | 0.8 |
| 1 | 2 | D6 | AM1248 azepane isomer | 2.5 |
| 1 | 2 | D7 | JWH 073 6-methoxyindole analog | 2.4 |
| 1 | 2 | E5 | 5-Fluoropentyl-3-pyridinoylindole | 2.3 |
| 1 | 2 | E7 | AM2201 N-(3-chloropentyl) isomer | 2.3 |
| 1 | 2 | E8 | 5-fluoro JWH 018 adamantyl analog | 2.1 |
| 1 | 3 | A3 | JWH 081-N-(cyclohexylmethyl) analog | 1.6 |
| 1 | 3 | A8 | THJ2201 | 2.2 |
| 1 | 3 | B9 | JWH 018 benzimidazole analog | 2.3 |
| 1 | 3 | C2 | JWH 018 benzimidazole analog | 3.1 |
| 1 | 3 | C3 | (+/-)-ORG 28611 | 1.5 |
| 1 | 3 | C6 | Mepirapim (hydrochloride) | 1.5 |
| 1 | 3 | D3 | JWH 019 N-(2-fluorohexyl) isomer | 1.9 |
| 1 | 3 | D4 | JWH 019 N-(3-fluorohexyl) isomer | 2.0 |
| 1 | 3 | D5 | JWH 019 N-(4-fluorohexyl) isomer | 2.0 |
| 1 | 3 | D6 | JWH 019 N-(5-fluorohexyl) isomer | 1.7 |
| 1 | 3 | D7 | JWH 019 N-(6-fluorohexyl) isomer | 1.2 |
| 1 | 3 | H3 | FUB-JWH 018 | 1.8 |
| 1 | 4 | A9 | 5-fluoro PY-PICA | 1.8 |
| 1 | 4 | B4 | 5-bromo THJ 018 | 2.4 |
| 1 | 4 | B5 | 5-chloro THJ 018 | 2.2 |
| 1 | 4 | C10 | IMMA | 2.3 |
| 1 | 4 | C4 | 5-fluoro BEPIRAPIM (hydrochloride) | 1.3 |
| 1 | 4 | C5 | 5-fluoro PY-PINACA (CRM) | 1.7 |
| 1 | 4 | D10 | JWH 018 N-(1,2-dimethylpropyl) isomer | 2.7 |
| 1 | 4 | D11 | JWH 018 2'-naphthyl isomer | 1.6 |
| 1 | 4 | D5 | JWH 018 adamantyl analog | 1.7 |
| 1 | 4 | D6 | JWH 200 2'-naphthyl isomer | 2.7 |
| 1 | 4 | D7 | JWH 018 N-(1,1-dimethylpropyl) isomer | 2.4 |
| 1 | 4 | D8 | JWH 018 N-(2,2-dimethylpropyl) isomer | 3.0 |
| 1 | 4 | D9 | JWH 018 N-(1-methylbutyl) isomer | 1.9 |
| 1 | 4 | E10 | JWH 073 2'-naphthyl isomer | 1.3 |
| 1 | 4 | E11 | JWH 073 2'-naphthyl-N-(2-methylpropyl) isomer | 1.5 |
| 1 | 4 | E2 | JWH 018 2'-naphthyl-N-(3-methylbutyl) isomer | 1.6 |
| 1 | 4 | E3 | JWH 018 2'-naphthyl-N-(1,2-dimethylbutyl) isomer | 1.2 |
| 1 | 4 | E4 | JWH 018 2'-naphthyl-N-(2,2-dimethylbutyl) isomer | 2.0 |
| 1 | 4 | E5 | JWH 018 2'-naphthyl-N-(1-methylbutyl) isomer | 1.0 |
| 1 | 4 | E6 | JWH 018 2'-naphthyl-N-(2-methylbutyl) isomer | 1.5 |
| 1 | 4 | E7 | JWH 073 N-(2-methylpropyl) isomer | 3.1 |
| 1 | 4 | E8 | JWH 073 N-(1-methylpropyl) isomer | 2.3 |
| 1 | 4 | E9 | JWH 073 N-(1,1-dimethylethyl) isomer | 1.2 |
| 1 | 4 | F10 | JWH 398 8-chloronaphthyl isomer | 2.7 |
| 1 | 4 | F11 | AM2201 N-(4-fluoropentyl) isomer | 1.3 |
| 1 | 4 | F2 | JWH 073 2'-naphthyl-N-(1-methylpropyl) isomer | 1.1 |
| 1 | 4 | F5 | JWH 398 2-chloronaphthyl isomer | 2.6 |
| 1 | 4 | F6 | JWH 398 3-chloronaphthyl isomer (hydrate) | 1.6 |
| 1 | 4 | F7 | JWH 398 5-chloronaphthyl isomer | 1.7 |
| 1 | 4 | F8 | JWH 398 6-chloronaphthyl isomer | 1.5 |
| 1 | 4 | F9 | JWH 398 7-chloronaphthyl isomer | 2.2 |
| 1 | 4 | G10 | JWH 210 2-ethylnaphthyl isomer | 2.5 |
| 1 | 4 | G11 | JWH 210 3-ethylnaphthyl isomer | 1.6 |
| 1 | 4 | G2 | AM2201 N-(3-fluoropentyl) isomer | 2.3 |
| 1 | 4 | G3 | AM2201 N-(2-fluoropentyl) isomer | 2.4 |
| 1 | 4 | G4 | JWH 122 2-methylnaphthyl isomer | 2.9 |
| 1 | 4 | G5 | JWH 122 3-methylnaphthyl isomer | 1.8 |
| 1 | 4 | G6 | JWH 122 5-methylnaphthyl isomer | 1.9 |
| 1 | 4 | G7 | JWH 122 6-methylnaphthyl isomer | 1.7 |
| 1 | 4 | G8 | JWH 122 7-methylnaphthyl isomer | 2.1 |
| 1 | 4 | G9 | JWH 122 8-methylnaphthyl isomer | 2.6 |
| 1 | 4 | H10 | JWH 081 7-methoxynaphthyl isomer | 2.0 |
| 1 | 4 | H11 | AM1220 | 3.4 |
| 1 | 4 | H2 | JWH 210 5-ethylnaphthyl isomer | 1.6 |
| 1 | 4 | H3 | JWH 210 6-ethylnaphthyl isomer | 1.5 |
| 1 | 4 | H4 | JWH 210 7-ethylnaphthyl isomer | 1.7 |
| 1 | 4 | H5 | JWH 210 8-ethylnaphthyl isomer | 2.3 |
| 1 | 4 | H6 | JWH 081 2-methoxynaphthyl isomer | 2.5 |
| 1 | 4 | H7 | JWH 081 3-methoxynaphthyl isomer | 1.7 |
| 1 | 4 | H8 | JWH 081 5-methoxynaphthyl isomer | 1.1 |
| 1 | 4 | H9 | JWH 081 6-methoxynaphthyl isomer | 1.4 |
| 1 | 5 | A10 | JWH 180 | 2.6 |
| 1 | 5 | A11 | MAM2201 | 2.1 |
| 1 | 5 | A2 | JWH 022 | 2.7 |
| 1 | 5 | A3 | JWH 011 | 2.2 |
| 1 | 5 | A4 | JWH 073 4-methylnaphthyl analog | 2.7 |
| 1 | 5 | A5 | AM1235 | 1.5 |
| 1 | 5 | A7 | JWH 072 | 2.8 |
| 1 | 5 | A8 | JWH 424 | 2.5 |
| 1 | 5 | A9 | WIN 54,461 | 1.4 |
| 1 | 5 | B5 | MN-25-2-methyl derivative | 1.4 |
| 1 | 5 | C5 | F2201 | 2.4 |
| 1 | 5 | D11 | Pravadoline | 1.4 |
| 1 | 5 | E2 | AM630 | 1.3 |
| 2 | 1 | A10 | RCS-8 | 2.7 |
| 2 | 1 | A7 | JWH 251 | 2.4 |
| 2 | 1 | B11 | JWH 201 | 2.9 |
| 2 | 1 | C2 | JWH 302 | 3.4 |
| 2 | 1 | D11 | JWH 203 4-chlorophenyl isomer | 2.8 |
| 2 | 1 | D7 | RCS-8 4-methoxy isomer | 3.0 |
| 2 | 1 | D8 | RCS-8 3-methoxy isomer | 3.1 |
| 2 | 1 | E2 | JWH 203 3-chlorophenyl isomer | 2.5 |
| 2 | 1 | G5 | STS-135 | 2.2 |
| 2 | 2 | B3 | AKB48 N-(5-fluoropentyl) analog | 1.4 |
| 2 | 2 | C11 | JWH 167 | 2.6 |
| 2 | 2 | C4 | JWH 250 | 2.4 |
| 2 | 2 | D8 | BB-22 | 3.2 |
| 2 | 2 | D9 | 5-fluoro NNEI | 1.4 |
| 2 | 2 | E10 | BB-22 4-hydroxyquinoline isomer | 5.4 |
| 2 | 2 | E11 | BB-22 5-hydroxyquinoline isomer | 1.9 |
| 2 | 2 | E6 | 5-fluoro NNEI 2'-naphthyl isomer | 1.2 |
| 2 | 2 | E9 | BB-22 3-hydroxyquinoline isomer | 1.6 |
| 2 | 2 | F10 | PB-22 4-hydroxyquinoline isomer | 5.0 |
| 2 | 2 | F11 | PB-22 5-hydroxyquinoline isomer | 2.3 |
| 2 | 2 | F2 | BB-22 6-hydroxyquinoline isomer | 1.3 |
| 2 | 2 | F3 | BB-22 7-hydroxyquinoline isomer | 3.0 |
| 2 | 2 | F4 | 5-fluoro PB-22 3-hydroxyquinoline isomer | 2.2 |
| 2 | 2 | F5 | 5-fluoro PB-22 4-hydroxyquinoline isomer | 2.5 |
| 2 | 2 | F6 | 5-fluoro PB-22 5-hydroxyquinoline isomer | 2.1 |
| 2 | 2 | F7 | 5-fluoro PB-22 6-hydroxyquinoline isomer | 3.4 |
| 2 | 2 | F8 | 5-fluoro PB-22 7-hydroxyquinoline isomer | 4.3 |
| 2 | 2 | G10 | 5-fluoro PB-22 N-(2-fluoropentyl) isomer | 2.5 |
| 2 | 2 | G11 | 5-fluoro PB-22 N-(3-fluoropentyl) isomer | 3.0 |
| 2 | 2 | G2 | PB-22 6-hydroxyquinoline isomer | 1.6 |
| 2 | 2 | G3 | PB-22 7-hydroxyquinoline isomer | 4.1 |
| 2 | 2 | G5 | BB-22 4-hydroxyisoquinoline isomer | 2.3 |
| 2 | 2 | G6 | BB-22 5-hydroxyisoquinoline isomer | 2.0 |
| 2 | 2 | G7 | BB-22 6-hydroxyisoquinoline isomer | 1.2 |
| 2 | 2 | G8 | BB-22 7-hydroxyisoquinoline isomer | 1.6 |
| 2 | 2 | H10 | PB-22 6-hydroxyisoquinoline isomer | 3.0 |
| 2 | 2 | H11 | PB-22 7-hydroxyisoquinoline isomer | 1.5 |
| 2 | 2 | H2 | 5-fluoro PB-22 N-(4-fluoropentyl) isomer | 2.4 |
| 2 | 2 | H3 | 5-fluoro PB-22 4-hydroxyisoquinoline isomer | 4.1 |
| 2 | 2 | H4 | 5-fluoro PB-22 5-hydroxyisoquinoline isomer | 2.4 |
| 2 | 2 | H5 | 5-fluoro PB-22 6-hydroxyisoquinoline isomer | 1.8 |
| 2 | 2 | H6 | 5-fluoro PB-22 7-hydroxyisoquinoline isomer | 1.2 |
| 2 | 2 | H7 | 5-fluoro PB-22 8-hydroxyisoquinoline isomer | 4.2 |
| 2 | 2 | H8 | PB-22 4-hydroxyisoquinoline isomer | 3.0 |
| 2 | 2 | H9 | PB-22 5-hydroxyisoquinoline isomer | 2.4 |
| 2 | 3 | A10 | MN-18 | 1.8 |
| 2 | 3 | A11 | THJ | 1.7 |
| 2 | 3 | A2 | PB-22 8-hydroxyisoquinoline isomer | 4.1 |
| 2 | 3 | A4 | NNEI 2'-naphthyl isomer | 1.1 |
| 2 | 3 | A9 | 5-fluoro MN-18 | 1.9 |
| 2 | 3 | B11 | SDB-006 | 2.5 |
| 2 | 3 | B2 | 5-fluoro THJ | 2.0 |
| 2 | 3 | B3 | JWH 018 8-quinolinyl carboxamide | 2.2 |
| 2 | 3 | B4 | AM2201 8-quinolinyl carboxamide | 1.8 |
| 2 | 3 | B6 | FUB-PB-22 | 1.9 |
| 2 | 3 | B7 | FDU-PB-22 | 1.6 |
| 2 | 3 | B8 | NNEI | 1.2 |
| 2 | 3 | C4 | NM2201 | 1.6 |
| 2 | 3 | C5 | 5-fluoro SDB-005 | 2.0 |
| 2 | 3 | C7 | SDB-005 | 2.0 |
| 2 | 3 | C8 | 5-fluoro SDB-006 | 1.4 |
| 2 | 3 | D11 | NPB-22 | 1.6 |
| 2 | 3 | E11 | SDB-006 N-phenyl analog | 1.4 |
| 2 | 3 | E2 | 5-fluoro NPB-22 | 1.4 |
| 2 | 3 | E6 | AKB48 N-(4-fluorobenzyl) analog | 2.3 |
| 2 | 3 | E7 | 3-CAF | 1.2 |
| 2 | 3 | E8 | 5-fluoro PCN | 0.5 |
| 2 | 3 | F10 | FUB-NPB-22 | 1.5 |
| 2 | 3 | F7 | CBL-018 | 1.6 |
| 2 | 3 | H11 | 5-fluoro CYPPICA | 1.0 |
| 2 | 3 | H6 | FDU-NNEI | 1.2 |
| 2 | 3 | H7 | NNEI 2'-indazole isomer | 1.4 |
| 2 | 4 | A11 | ATHPINACA isomer 1 | 1.0 |
| 2 | 4 | A7 | 5-chloro AKB48 | 1.2 |
| 2 | 4 | B11 | ATHPINACA isomer 2 | 1.1 |
| 2 | 4 | B6 | APINAC | 1.7 |
| 2 | 4 | B7 | 5-fluoro APINAC | 1.0 |
| 2 | 4 | D4 | JWH 203 | 1.9 |
| 2 | 4 | F3 | JWH 251 3-methylphenyl isomer | 2.0 |
| 2 | 4 | F4 | JWH 251 4-methylphenyl isomer | 1.9 |
| 2 | 5 | A6 | JWH 018 adamantyl carboxamide | 2.4 |
| 2 | 5 | D5 | 2-fluoro NNEI | 1.4 |
| 2 | 5 | D6 | 3-fluoro NNEI | 1.2 |
| 2 | 5 | D7 | 4-fluoro NNEI | 1.2 |
| 2 | 5 | F2 | STS-135 | 1.3 |
| 3 | 2 | E2 | MN-25 | 3.1 |
| 3 | 2 | E3 | ADB-FUBINACA | 1.6 |
| 3 | 2 | E4 | ADBICA | 2.5 |
| 3 | 3 | A5 | 5-fluoro AB-PINACA | 1.4 |
| 3 | 3 | A6 | 5-fluoro ADB-PINACA | 1.4 |
| 3 | 3 | A7 | 5-fluoro ADBICA | 1.4 |
| 3 | 3 | C10 | AB-PINACA N-(2-fluoropentyl) isomer | 1.3 |
| 3 | 3 | C11 | AB-PINACA N-(3-fluoropentyl) isomer | 1.2 |
| 3 | 3 | C9 | AB-CHMINACA | 2.8 |
| 3 | 3 | D2 | AB-PINACA N-(4-fluoropentyl) isomer | 1.4 |
| 3 | 3 | D8 | AMB | 1.9 |
| 3 | 3 | D9 | 5-fluoro AMB | 1.5 |
| 3 | 3 | E10 | MMB2201 | 1.0 |
| 3 | 3 | E4 | PF-03550096 | 1.1 |
| 3 | 3 | E9 | MMB018 | 1.2 |
| 3 | 3 | F3 | MDMB-CHMINACA | 2.8 |
| 3 | 3 | F4 | PX 1 | 1.5 |
| 3 | 3 | F5 | MA-CHMINACA | 2.9 |
| 3 | 3 | F6 | PX 2 | 1.7 |
| 3 | 3 | F8 | 5-fluoro ADB | 1.9 |
| 3 | 3 | F9 | MAB-CHMINACA | 3.3 |
| 3 | 3 | G10 | CUMYL-PICA | 1.1 |
| 3 | 3 | G11 | 5-fluoro CUMYL-PICA | 1.0 |
| 3 | 3 | G2 | MDMB-FUBINACA | 3.4 |
| 3 | 3 | G3 | MDMB-CHMICA | 3.0 |
| 3 | 3 | G5 | MO-CHMINACA | 2.8 |
| 3 | 3 | G6 | 5-fluoro-2-ADB-PINACA isomer 2 | 1.0 |
| 3 | 3 | G8 | APP-FUBINACA | 1.8 |
| 3 | 3 | G9 | CUMYL-THPINACA | 1.3 |
| 3 | 3 | H10 | APP-CHMINACA | 3.4 |
| 3 | 3 | H4 | 5-fluoro ADB-PINACA isomer 2 | 1.4 |
| 3 | 3 | H5 | EMB-FUBINACA | 3.0 |
| 3 | 3 | H8 | AB-CHMINACA 2'-indazole isomer | 1.1 |
| 3 | 3 | H9 | APP-PICA | 1.6 |
| 3 | 4 | A10 | MDMB-FUBICA | 1.5 |
| 3 | 4 | A2 | 5-fluoro CUMYL-PINACA (CRM) | 1.7 |
| 3 | 4 | A3 | 5-fluoro AEB | 1.7 |
| 3 | 4 | A4 | MMB-CHMICA | 1.7 |
| 3 | 4 | A5 | AB-CHMICA | 2.4 |
| 3 | 4 | B10 | AB-BICA | 1.3 |
| 3 | 4 | B2 | 5-fluoro CUMYL-P7AICA | 1.3 |
| 3 | 4 | B8 | ADB-BINACA | 1.8 |
| 3 | 4 | B9 | AB-FUBICA | 1.7 |
| 3 | 4 | C2 | MDMB-CHMCZCA | 1.7 |
| 3 | 4 | C3 | ADB-BICA | 1.9 |
| 3 | 4 | C6 | 4-fluoro ADB (CRM) | 2.5 |
| 3 | 4 | C7 | MMB-FUBICA | 1.5 |
| 3 | 4 | C8 | 4-cyano CUMYL-BUTINACA | 1.5 |
| 3 | 4 | C9 | 4-cyano CUMYL-BUTINACA isomer 2 | 1.4 |
| 3 | 5 | B10 | ADB-PINACA isomer 3 | 4.6 |
| 3 | 5 | B11 | AB-FUBINACA isomer 1 | 2.0 |
| 3 | 5 | B6 | AB-FUBINACA 3-fluorobenzyl isomer | 1.8 |
| 3 | 5 | B7 | AB-FUBINACA 2-fluorobenzyl isomer | 2.2 |
| 3 | 5 | B8 | ADB-PINACA isomer 1 | 2.1 |
| 3 | 5 | B9 | ADB-PINACA isomer 2 | 3.5 |
| 3 | 5 | C10 | JWH 200 analog 1 | 2.2 |
| 3 | 5 | C11 | MMB-FUBINACA | 2.5 |
| 3 | 5 | C2 | AB-FUBINACA isomer 2 | 1.4 |
| 3 | 5 | C3 | AB-FUBINACA isomer 5 | 2.2 |
| 3 | 5 | C4 | 5-fluoro ABICA | 1.4 |
| 3 | 5 | C6 | 5-chloro AB-PINACA | 2.0 |
| 3 | 5 | D2 | ADB-PINACA isomer 4 | 3.3 |
| 3 | 5 | D3 | 3-fluoro AMB | 1.8 |
| 3 | 5 | D4 | 4-fluoro AMB | 1.5 |
| 3 | 5 | F3 | AB-PINACA (CRM) | 2.4 |
| 3 | 5 | F4 | AB-FUBINACA (CRM) | 2.0 |
| 3 | 5 | F5 | ADB-PINACA (CRM) | 2.6 |
| 4 | 1 | A3 | (R)-AM1241 | 3.6 |
| 4 | 5 | E9 | AM1241 | 5.6 |
| 5 | 1 | F10 | A-834735 | 1.6 |
| 5 | 1 | G11 | A-796260 | 2.1 |
| 5 | 1 | G6 | XLR11 | 3.0 |
| 5 | 1 | H10 | XLR11 N-(2-fluoropentyl) isomer | 2.1 |
| 5 | 1 | H11 | XLR11 N-(3-fluoropentyl) isomer | 2.2 |
| 5 | 1 | H7 | XLR11 N-(4-pentenyl) analog | 2.1 |
| 5 | 1 | H9 | AB-005 | 0.9 |
| 5 | 2 | A11 | UR-144 N-(5-bromopentyl) analog | 1.8 |
| 5 | 2 | A2 | XLR11 N-(4-fluuoropentyl) isomer | 1.7 |
| 5 | 2 | A6 | AB-005 azepane isomer | 1.4 |
| 5 | 2 | A8 | UR-144 N-(5-chloropentyl) analog | 2.2 |
| 5 | 2 | B5 | UR-144 N-(2-chloropentyl) analog | 2.1 |
| 5 | 2 | B9 | URB447 | 3.2 |
| 5 | 2 | B7 | UR-144 N-(4-chloropentyl) analog | 2.4 |
| 5 | 2 | C8 | A-836339 | 2.4 |
| 5 | 2 | D10 | UR-144 N-heptyl analog | 2.3 |
| 5 | 3 | B10 | FAB-144 | 1.9 |
| 5 | 3 | B5 | XLR12 | 2.4 |
| 5 | 3 | E3 | FUB-144 | 2.8 |
| 5 | 3 | F2 | M-144 | 2.3 |
| 5 | 3 | G4 | Azidoindoline 1 | 2.4 |
| 5 | 5 | B2 | UR-144 N-(5-methylhexyl) analog | 1.7 |
| 5 | 5 | E11 | UR-144 | 2.5 |
| 6 | 1 | C10 | JWH 369 | 2.8 |
| 6 | 1 | C11 | JWH 368 | 2.4 |
| 6 | 1 | C4 | JWH 307 | 2.5 |
| 6 | 1 | C6 | JWH 031 | 2.7 |
| 6 | 1 | C7 | JWH 145 | 2.6 |
| 6 | 1 | C8 | JWH 147 | 2.5 |
| 6 | 1 | C9 | JWH 370 | 2.8 |
| 6 | 1 | D2 | JWH 309 | 1.8 |
| 6 | 1 | D3 | JWH 030 | 2.7 |
| 6 | 1 | H4 | JWH 031 2'-isomer | 1.9 |
| 6 | 1 | H5 | JWH 030 2-naphthoyl isomer | 1.9 |
| 6 | 2 | B9 | URB447 | 3.4 |
| 6 | 3 | G7 | 5-fluoro-3,5-AB-PFUPPYCA | 1.3 |
| 6 | 4 | A8 | 5,3-AB-CHMFUPPYCA | 1.1 |
| 6 | 4 | B3 | 5-fluoro-3,5-ADB-PFUPPYCA | 2.3 |
| 7 | 1 | E10 | (+)-CP 47,497 | 3.1 |
| 7 | 1 | E11 | (+/-)-CP 47,497 | 1.1 |
| 7 | 1 | E5 | CP 47,497-para-quinone analog | 2.8 |
| 7 | 1 | E9 | (+/-)-CP 47,497-C8-homolog | 1.7 |
| 7 | 1 | F2 | ()-CP 47,497 | 0.8 |
| 7 | 1 | F3 | (+/-)3-epi CP 47,497-C8- homolog | 1.4 |
| 7 | 1 | F4 | (+/-)3-epi CP 47,497 | 3.2 |
| 7 | 2 | B8 | (+/-)-CP 55,940 | 0.2 |
| 7 | 2 | C2 | (+)-CP 55,940 | 0.9 |
| 7 | 2 | C6 | (+/-)5-epi CP 55,940 | 0.3 |
| 7 | 4 | C11 | HU-210 | 0.2 |
| 7 | 4 | D2 | ()-CP 55,940 | 1.6 |
| 7 | 4 | D3 | HU-308 | 1.4 |
| 7 | 5 | B3 | CP 47,497-C6-homolog | 1.8 |
| 7 | 5 | B4 | CP 47,497-C9-homolog | 0.5 |
| 7 | 5 | E4 | JP104 | 1.9 |
| N/A | 1 | A11 | MDA 77 | 1.6 |
| N/A | 1 | A5 | MDA 19 | 2.3 |
| N/A | 1 | A6 | AM694 | 2.9 |
| N/A | 1 | B2 | KM 233 | 0.9 |
| N/A | 1 | C3 | (+/-)-WIN 55,212 (mesylate) | 0.3 |
| N/A | 1 | D6 | AM2201 2'-naphthyl isomer | 2.2 |
| N/A | 1 | E3 | AM694 4-iodo isomer | 1.5 |
| N/A | 1 | E4 | AM694 3-iodo isomer | 2.3 |
| N/A | 1 | F11 | JWH 175 | 1.6 |
| N/A | 1 | F5 | 3,4-MDMA methylene homolog (hydrochloride) | 1.1 |
| N/A | 1 | F9 | JWH 249 | 2.3 |
| N/A | 1 | H3 | JWH 176 | 1.4 |
| N/A | 1 | H8 | SER-601 | 0.9 |
| N/A | 2 | B10 | CB-86 | 2.1 |
| N/A | 2 | B11 | AM3102 | 1.7 |
| N/A | 2 | D5 | 1-(4-Methoxyphenyl) piperazine (hydrochloride) | 1.1 |
| N/A | 2 | G4 | LY2183240 2'-isomer | 1.5 |
| N/A | 3 | D10 | EG 018 (EG 018 is an analog of JWH 018 that has a benzene ring attached to the aminoalkylindole group, positioned adjacent to the naphthoyl group.) | 1.3 |
| N/A | 3 | E5 | PSB-SB1202 | 1.9 |
| N/A | 3 | F11 | EG2201 ( EG2201 is an analog of AM2201 that has a benzene ring attached to the aminoalkylindole group, positioned adjacent to the naphthoyl group.) | 1.3 |
| N/A | 4 | A6 | Flurazepam (CRM) (a benzodiazepine; acts as a partial agonist of the benzodiazepine site on GABAA receptors, potentiating the action of GABA with an EC50 value of 930 nM in neuronal chick spinal cord cultures. Through this signaling mechanism, flurazepam produces anxiolytic and sedative properties.) | 1.6 |
| N/A | 5 | C7 | PTI-1 (hydrochloride) | 0.9 |
| N/A | 5 | C8 | MCHB-1 | 0.9 |
| N/A | 5 | C9 | PTI-2 (hydrochloride) | 0.8 |
| N/A | 5 | D10 | HU-211 | 1.0 |
| N/A | 5 | D8 | JWH 133 | 1.9 |
| N/A | 5 | D9 | HU-331 | 0.3 |
| N/A | 5 | E10 | CB-13 | 2.1 |
| N/A | 5 | E3 | URB602 | 1.7 |
| N/A | 5 | E5 | LY2183240 | 1.5 |
| N/A | 5 | E6 | (+)-WIN 55,212-2 (mesylate) | 0.2 |
| N/A | 5 | E7 | CB-25 | 1.2 |
| N/A | 5 | E8 | CB-52 | 1.4 |
